# EXD2 Protects Stressed Replication Forks and Is Required for Cell Viability in the Absence of BRCA1/2

**DOI:** 10.1016/j.molcel.2019.05.026

**Published:** 2019-08-08

**Authors:** Jadwiga Nieminuszczy, Ronan Broderick, Marina A. Bellani, Elizabeth Smethurst, Rebekka A. Schwab, Veronica Cherdyntseva, Theodora Evmorfopoulou, Yea-Lih Lin, Michal Minczuk, Philippe Pasero, Sarantis Gagos, Michael M. Seidman, Wojciech Niedzwiedz

**Affiliations:** 1 The Institute of Cancer Research, London, UK; 2 Laboratory of Molecular Gerontology, National Institute on Aging, Baltimore, MD, USA; 3 Institute of Molecular Medicine, Oxford, UK; 4 Laboratory of Genetics, Biomedical Research Foundation of the Academy of Athens, Athens, Greece; 5 Institut de Génétique Humaine, CNRS, Université de Montpellier, Montpellier, France; 6 MRC Mitochondrial Biology Unit, University of Cambridge, Cambridge, UK

**Keywords:** EXD2, EXDL2, DNA replication, fork regression, BRCA1, BRCA2

## Abstract

Accurate DNA replication is essential to preserve genomic integrity and prevent chromosomal instability-associated diseases including cancer. Key to this process is the cells’ ability to stabilize and restart stalled replication forks. Here, we show that the EXD2 nuclease is essential to this process. EXD2 recruitment to stressed forks suppresses their degradation by restraining excessive fork regression. Accordingly, EXD2 deficiency leads to fork collapse, hypersensitivity to replication inhibitors, and genomic instability. Impeding fork regression by inactivation of SMARCAL1 or removal of RECQ1’s inhibition in *EXD2*^−/−^ cells restores efficient fork restart and genome stability. Moreover, purified EXD2 efficiently processes substrates mimicking regressed forks. Thus, this work identifies a mechanism underpinned by EXD2’s nuclease activity, by which cells balance fork regression with fork restoration to maintain genome stability. Interestingly, from a clinical perspective, we discover that EXD2’s depletion is synthetic lethal with mutations in BRCA1/2, implying a non-redundant role in replication fork protection.

## Highlights

• EXD2 is required for cell survival in response to replicative stress
• EXD2 protects replication forks from over resection by counteracting fork reversal
• EXD2 is synthetic lethal with the deficiency in *BRCA1/2* genes

## Graphical Abstract

**Figure:**
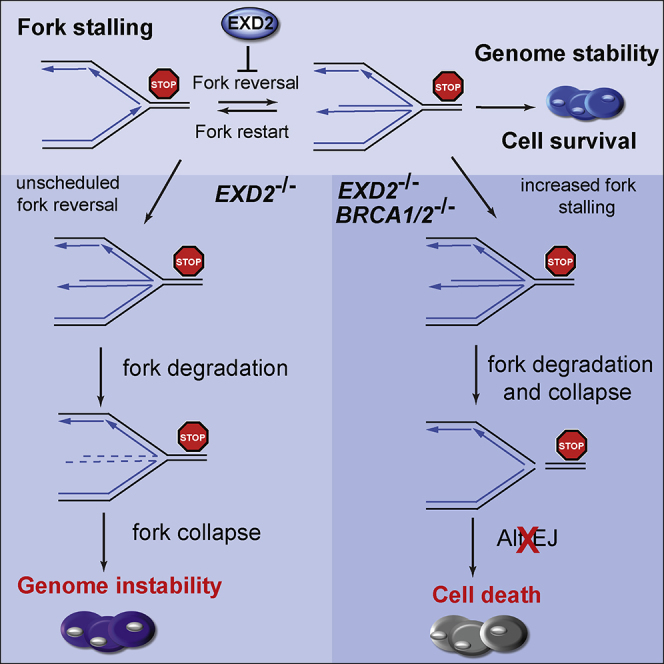

## Teaser

Nieminuszczy et al. identify a key function of the EXD2 nuclease in DNA replication and alternative end-joining. EXD2 localizes to replication forks and promotes their stabilization by counteracting fork regression. Loss of EXD2 results in sensitivity to replicative inhibitors, degradation of regressed forks, and compromises survival of BRCA1/2-deficient tumors.

## Introduction

Faithful duplication of the genome during cell division ensures accurate transmission of genetic information to daughter cells. This process relies on the replication of the entire genomic DNA during S-phase by thousands of replication forks. However, replication fork stability is constantly challenged by damage to the DNA template or progression through chromosomal regions that are inherently difficult to replicate (Bhowmick and Hickson, 2017, Gaillard et al., 2015, Kolinjivadi et al., 2017a). These blockades can collapse replication forks, contributing to tumor progression by driving chromosomal instability (Burrell et al., 2013, Hills and Diffley, 2014, Jeggo et al., 2016, Kass et al., 2016, Zeman and Cimprich, 2014). To counteract this threat, cells possess mechanisms to protect stalled replication forks, the most important of which is the replication fork protection pathway. This surveillance pathway, underpinned by the ATR kinase, ensures inhibition of cell-cycle progression, suppression of late origin firing, and restart of stalled replication forks (Jackson and Bartek, 2009, Kass et al., 2016, O’Driscoll, 2012). Together, these events ensure the faithful completion of DNA synthesis (Cimprich and Cortez, 2008, Neelsen and Lopes, 2015, Zeman and Cimprich, 2014). The importance of these responses is highlighted by several cancer-predisposing human diseases caused by mutations in various proteins contributing to replication fork stability (e.g., Seckel, Bloom, Werner, or Fanconi anemia syndromes).

Recently, fork reversal has emerged as a key mechanism protecting stressed replication forks (Neelsen and Lopes, 2015, Ray Chaudhuri et al., 2012, Zellweger et al., 2015). This process involves regression of the fork, mediated by several SNF2-family fork remodelers, and the formation of a 4-way junction. Once the blockade is removed, regressed forks remain capable of resuming DNA synthesis. The restart process is mediated by the RECQ1 helicase, which has the ability to migrate and resolve these 4-way structures (Berti et al., 2013, Neelsen and Lopes, 2015). Interestingly, in bacteria and yeast, a controlled nucleolytic processing of regressed nascent strands stabilizes stalled forks and prevents their reversal (Hu et al., 2012, Yeeles et al., 2013). Whether a similar mechanism exists in mammalian cells is currently unclear. Nevertheless, the ability to “resolve” regressed replication forks is crucial for genome stability, because the unregulated activity of fork remodelers induces degradation of nascent DNA and drives chromosomal instability (Couch et al., 2013, Dungrawala et al., 2017, Thangavel et al., 2015).

Elegant work from several laboratories indicated that the BRCA1/2 proteins protect nascent DNA at reversed forks (Kolinjivadi et al., 2017b, Lemaçon et al., 2017, Mijic et al., 2017, Schlacher et al., 2011, Taglialatela et al., 2017). The fork protection function of BRCA1/2 is RAD51-dependent, as cells depleted for RAD51 or lacking the RAD51 stabilizing factor BOD1L, display extensive nascent DNA degradation (Hashimoto et al., 2010, Higgs et al., 2015, Schlacher et al., 2011). Fork protection also requires components of the Fanconi anemia pathway, which cooperate with BRCA1/2 in suppressing nascent DNA degradation (Schlacher et al., 2011). More recently, several nucleases (i.e., MRE11, DNA2, EXO1, and WRN) have also been implicated in supporting damaged forks (Iannascoli et al., 2015, Karanja et al., 2014, Lemaçon et al., 2017, Thangavel et al., 2015). Consequently, defective fork processing by these enzymes leads to fork collapse, increased genomic instability, and contributes to the development of chemotherapy resistance (Chaudhuri et al., 2016, Neelsen and Lopes, 2015).

Despite extensive research, it is not fully understood how cells maintain genome stability at sites of stalled DNA replication and how the different nucleases and fork remodelers function to balance fork regression with fork restart to restore the replication program. Because replication stress drives tumorigenesis and restoration of fork protection in cancer cells facilitates the development of chemotherapy resistance, it is crucial to understand these mechanisms (Berti and Vindigni, 2016, Kolinjivadi et al., 2017a, Yeeles et al., 2013).

Here, we show that the EXD2 nuclease, a protein recently shown to promote genome stability in *Drosophila* (Cox et al., 2007) and human cell lines (Biehs et al., 2017, Broderick et al., 2016, Smogorzewska et al., 2010), is a component of the replication fork protection pathway. Accordingly, EXD2 is recruited to stalled replication forks and cells lacking EXD2 or expressing a nuclease-dead version of the protein display high levels of replication-associated genome instability. Mechanistically, we show that EXD2 acts to counteract fork reversal and this activity is critical for suppression of uncontrolled degradation of nascent DNA and efficient fork restart. In line with this, *EXD2*^−/−^ cells accumulate regressed replication forks and restricting fork regression by silencing SMARCAL1 or removal of PARP1-dependent inhibition of RECQ1 suppresses their degradation and allows for efficient fork restart in these cells. Purified EXD2 can process synthetic fork-like structures *in vitro*, and *in vivo* its nuclease activity acts to suppress the collapse of terminally regressed forks. Unexpectedly, we also discover that depletion of EXD2 confers a synthetic lethal interaction with BRCA1/2, suggesting a non-redundant function between these repair factors. Taken together, our findings uncover a previously unknown role for EXD2 in the replication stress response and also identifies EXD2 as a potential druggable target for cancer therapy.

## Results

### EXD2 Is Recruited to Replication Forks following Replication Stress

**Figure 1:**
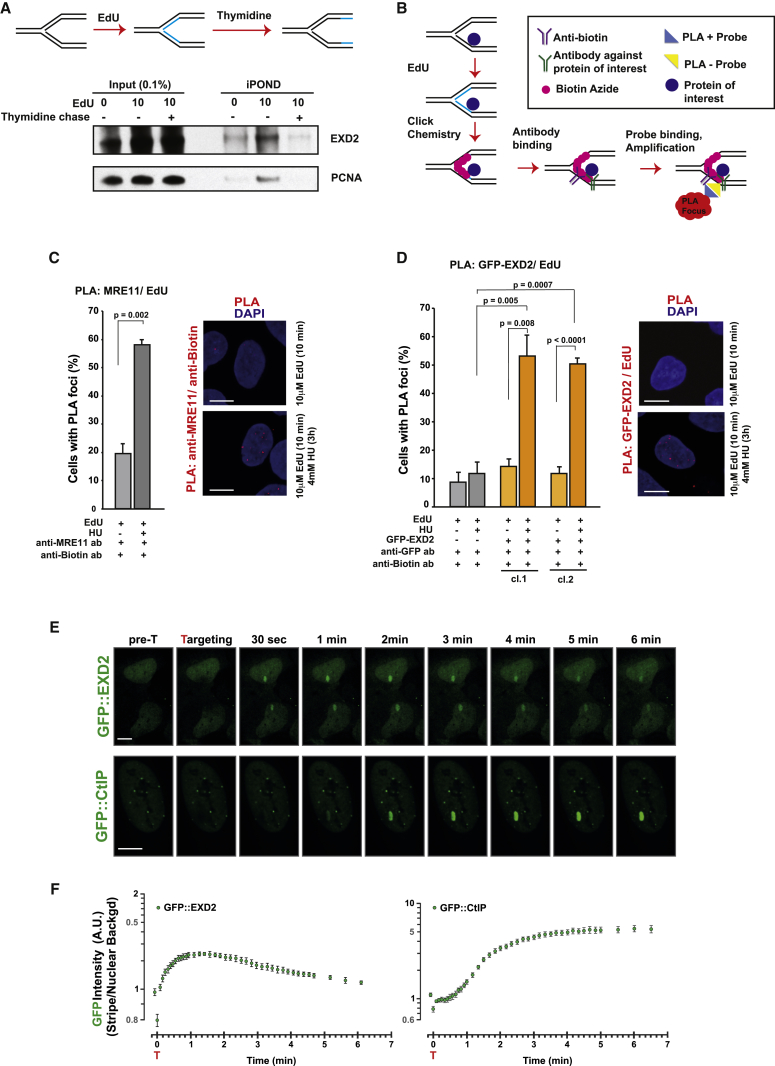
EXD2 Is Recruited to Stressed Replication Forks (A) Western blot of iPOND samples. Thymidine chase analysis illustrates that EXD2 specifically associates with the replisome. PCNA acts as a control. (B) Schematic of the proximity ligation assay (PLA) employed to detect colocalization of target proteins with nascent DNA. (C) Percentage of cells with MRE11/biotin PLA foci (mean ± SEM, n = 3 independent experiments, t test). Right: representative images of PLA foci (red), DAPI acts as a nuclear counterstain. Scale bar, 10 μm. (D) Percentage of cells with GFP/biotin PLA foci (mean ± SEM, n = 3 independent experiments, t test) in U2OS control cells and U2OS cells expressing GFP-EXD2. Right: representative images of PLA foci (red), DAPI acts as a nuclear counterstain. Scale bar, 10 μm. (E) Laser microirradiation induces rapid redistribution of GFP-EXD2 to damaged chromatin; representative images showing GFP-EXD2 accumulation at laser-generated DNA lesions. GFP-CtIP was used as a positive control. Scale bar, 10 μm. (F) Quantification of GFP-EXD2 (left panel) and GFP-CtIP (right panel) recruitment kinetics (intensity versus time) to laser-generated DNA lesions (mean ± SE, n ≥ 10 cells from 2 independent experiments).

Recently, we have employed isolation of proteins on nascent DNA (iPOND) coupled with mass spectrometry to identify factors recruited to stalled replication forks (Higgs et al., 2015). This analysis identified EXD2, as a factor recruited to replication forks (Figure S1A). We confirmed these results by western blotting (Figure S1B) (Coquel et al., 2018). To test if EXD2 associates specifically with replication forks, we performed an iPOND analysis coupled with a thymidine-chase. This revealed that the abundance of EXD2 decreased upon the chase with thymidine (Figure 1A) as observed previously for PCNA (Sirbu et al., 2011). To further verify EXD2’s association with newly replicated DNA, we combined EdU labeling with the proximity-ligation assay (PLA) to gauge the proximity of proteins with labeled nascent DNA (Higgs et al., 2015, Taglialatela et al., 2017) (Figures 1B and S1C). To this end, U2OS cells stably expressing GFP-EXD2 (Figure S1D) were labeled with EdU and subsequently treated with hydroxyurea (HU) followed by PLA to detect protein association with biotin-labeled nascent DNA. First, we validated this approach by testing the co-localization of MRE11 with nascent DNA after replication stress. As expected, MRE11 was significantly enriched following HU treatment (Figure 1C), consistent with its role at the stressed forks (Costanzo, 2011, Hashimoto et al., 2010, Taglialatela et al., 2017). Importantly, we could also readily detect nuclear PLA signal for EXD2 in cells treated with HU (Figure 1D), which was significantly enriched compared to untreated and control samples. To ascertain that this phenotype is not restricted to the GFP tag or its position, we repeated these experiments using U2OS cells expressing FLAG-tagged EXD2 (Broderick et al., 2016) and C-terminally GFP-tagged EXD2 (Figures S1E and S1F), confirming the specificity of its nuclear co-localization with stalled forks. Moreover, time-dependent analysis of EXD2 recruitment to stalled forks revealed similar kinetics to those of MRE11 (Figures S2A–S2D). Next, to gain further insight into the dynamics of EXD2 recruitment to DNA lesions, we employed laser micro-irradiation combined with live cell imaging (Suhasini et al., 2013). This analysis revealed that GFP-EXD2 is rapidly recruited to laser-generated DNA damage, with faster kinetics than those of GFP-CtIP (Figures 1E and 1F; ,Video S1), underscoring its early role in the DNA repair processes. Taken together, this data suggest that EXD2 is rapidly recruited to damaged chromatin and associates with sites of DNA replication.

Video S1. GFP-EXD2 Accumulation at Laser-Generated DNA Lesions, Related to Figures 1E and 1F

### EXD2 Promotes Global Replication Fork Dynamics in Response to Replicative Stress

**Figure 2:**
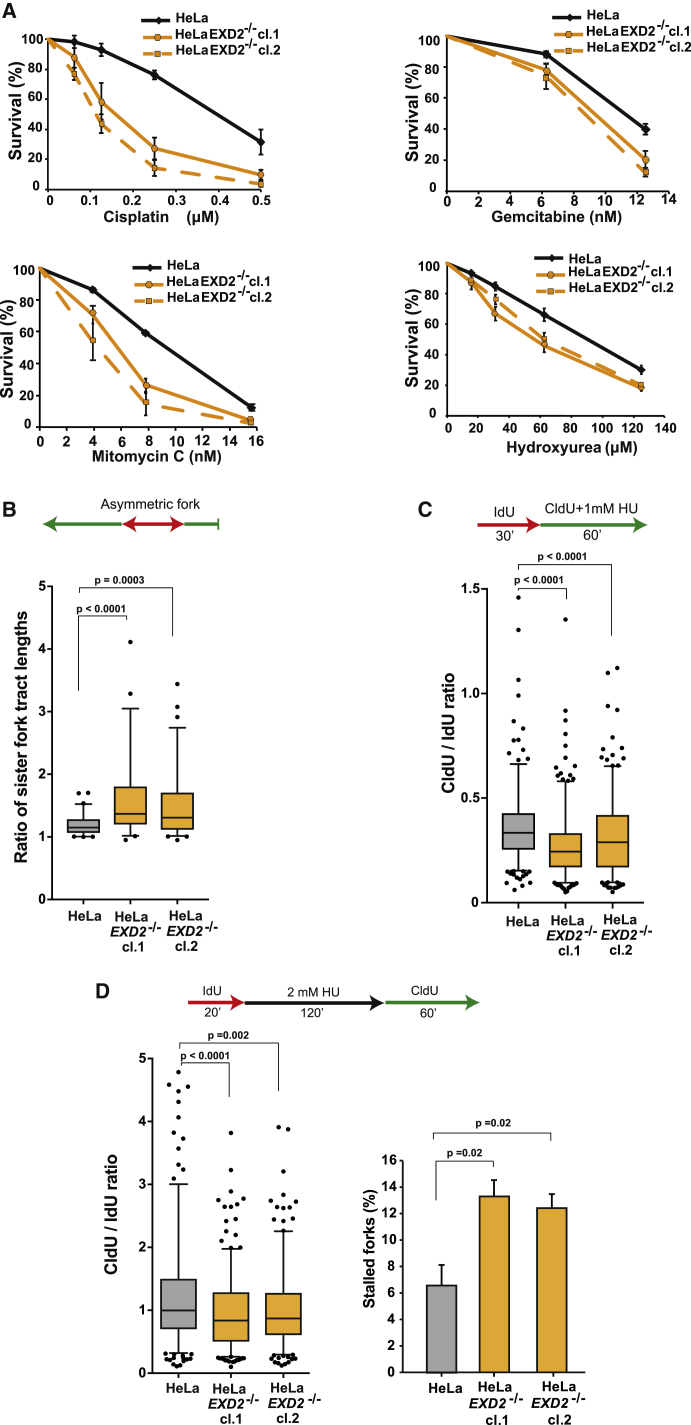
EXD2 Promotes Global Replication Fork Dynamics in Response to Replicative Stress (A) Survival of HeLa control and HeLa *EXD2*^−/−^ cells treated with the indicated doses of cisplatin, gemcitabine, mitomycin C, or hydroxyurea (mean ± SEM, n = 3 independent experiments). (B) Boxplot of CldU tract length ratios of associated sister forks from HeLa WT and *EXD2*^−/−^ cells. (5–95 percentile, n ≥ 60 sister fork pairs pooled from 3 independent experiments, Mann-Whitney). (C) Boxplot of CldU/IdU tract ratios of HeLa WT and *EXD2*^−/−^ cells treated with 1 mM HU (5–95 percentile, n ≥ 300 tracts pooled from 3 independent experiments, Mann-Whitney). (D) Boxplot of CldU/IdU tract ratios of HeLa WT and *EXD2*^−/−^ cells (left panel) and quantification of the percentage of stalled forks (red only tracts) in HeLa WT and *EXD2*^−/−^ cells (right panel) (5–95 percentile, n ≥ 300 tracts pooled from 3 independent experiments Mann-Whitney [left panel]; n = 3 independent experiments, t test [right panel]).

To shed light on the role of EXD2 during DNA replication, we analyzed the survival of *EXD2*^−/−^ cells exposed to agents that impede fork progression. Loss of EXD2 sensitizes cells not only to MMC, CPT, and MMS as reported (Broderick et al., 2016, Smogorzewska et al., 2010) but also other drugs capable of blocking fork progression, i.e., cisplatin, gemcitabine, and HU (Figures 2A, S2E, and S2F), suggesting a general role in mitigating replicative stress.

**Figure 3:**
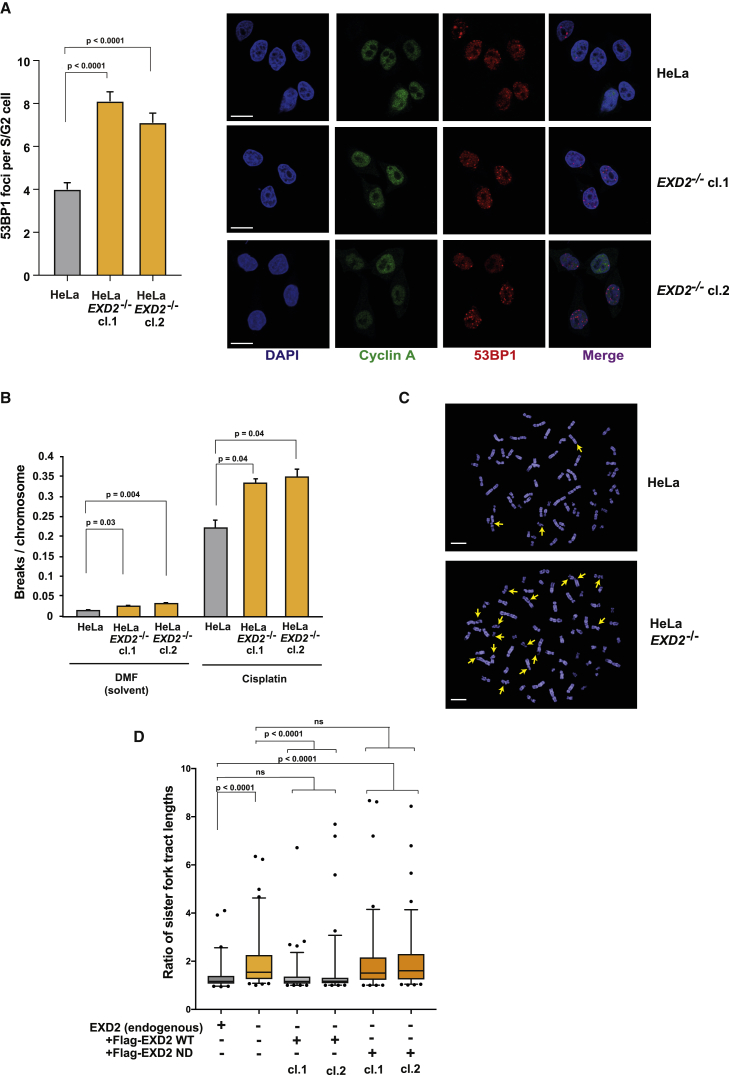
EXD2’s Nuclease Activity Is Required to Suppress Replication Fork Collapse (A) Quantification of the frequency of 53BP1 foci in HeLa WT and *EXD2*^−/−^ S/G2 cells and representative images. Cyclin A (green) acts as a marker for S/G2 cells, DAPI acts as a nuclear stain (mean ± SEM, n = 3 independent experiments, Mann-Whitney). Scale bar, 10 μm. (B) Quantification of the frequency of chromosomal aberrations from mitotic spreads from HeLa WT and *EXD2*^−/−^ cells (mean ± SEM, n = 75 metaphase spreads pooled from 3 independent experiments, t test). (C) Representative images of metaphase spreads from B). Arrows indicate chromatid breaks. Scale bar, 6.5 μm. (D) Boxplot of CldU tract length ratios of associated sister forks from HeLa WT, *EXD2*^−/−^, and *EXD2*^−/−^ cells complemented with either Flag-EXD2 WT or Flag-EXD2 nuclease dead (ND) mutant protein (5–95 percentile, n ≥ 60 sister fork pairs pooled from 3 independent experiments, Mann-Whitney).

To address the mechanism by which EXD2 promotes DNA replication, we used the DNA fiber assay to visualize individual replication forks. First, we quantified replication fork asymmetry in unchallenged cells (Schwab et al., 2015). We observed a significant increase in sister fork asymmetry in HeLa *EXD2*^−/−^ cells (Figure 2B), suggesting increased rates of fork stalling or collapse. Consistently, transient exposure of EXD2-deficient cells to HU impeded replication fork dynamics (Figure 2C) as well as restart of stressed forks (Figure 2D). Moreover, forks that were able to recover DNA synthesis did so with a significant delay (Figure 2D). To determine the fate of stalled replication forks, we analyzed the formation of 53BP1 foci, a marker of double strand break (DSB) induction. This analysis revealed that loss of EXD2 leads to a significant increase in the number of 53BP1 foci per S/G2-cell, likely due to a wide-spread fork collapse resulting in the formation of DSBs (Figure 3A). Consistent with this, *EXD2*^−/−^ cells displayed high levels of chromatid-type aberrations associated with collapsed replication forks (Figures 3B and 3C). Finally, because EXD2 is a 3′-5′ exonuclease (Broderick et al., 2016), we tested the ability of HeLa *EXD2*^−/−^ cells complemented with either wild type (WT) or nuclease-dead EXD2 to restore the normal replication program (i.e., fork symmetry). Only cells expressing WT EXD2, but not the nuclease-dead mutant, are capable of restoring efficient fork progression (Figures 3D and S2G). Consistent with this, purified EXD2 can process synthetic fork-like structures *in vitro* (Figure S2H) suggesting that its nuclease activity facilitates fork repair. Collectively, these data indicate that EXD2 is a component of the replication fork protection pathway supporting efficient DNA synthesis and suppressing genome instability.

### Loss of EXD2 Leads to Mitotic Abnormalities Associated with Under-Replicated DNA

**Figure 4:**
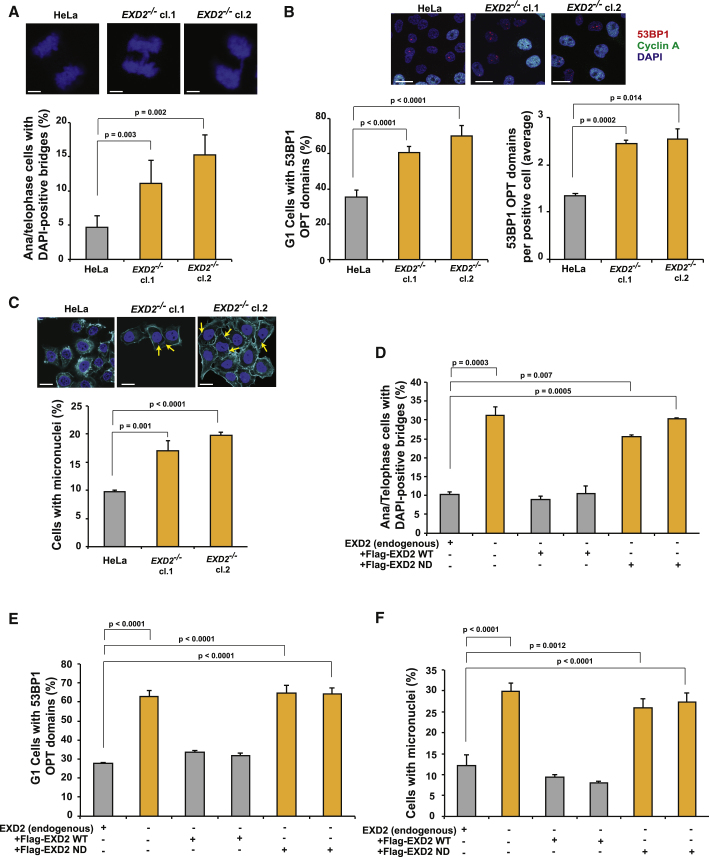
Loss of EXD2 Leads to Mitotic Abnormalities Associated with Under-Replicated DNA (A) Quantification of the HeLa WT and *EXD2*^−/−^ anaphase or telophase cells showing DAPI-positive bridges (mean ± SEM, n = 3 independent experiments, chi-square). Scale bar, 10 μm. (B) Quantification of HeLa WT and *EXD2*^−/−^ G1 cells with 53BP1 OPT domains in G1 cells (left panel). Quantification of the number of 53BP1 OPT domains per positive cell in HeLa WT and *EXD2*^−/−^ cells (right panel) and representative images (mean ± SEM, n = 3 independent experiments, chi-square). Scale bar, 20 μm. (C) Quantification of HeLa WT and *EXD2*^−/−^ cells showing MN and representative images. Phalloidin acts as a cytosolic marker (mean ± SEM, n = 3 independent experiments, chi-square). Scale bar, 20 μm. (D) Quantification of HeLa WT, *EXD2*^−/−^, and *EXD2*^−/−^ cells complemented with either Flag-EXD2 WT or Flag-EXD2 nuclease dead (ND) mutant protein for anaphase or telophase cells showing DAPI-positive bridges (mean ± SEM, n = 3 independent experiments, chi-square). (E) Quantification of HeLa WT, *EXD2*^−/−^, and *EXD2*^−/−^ cells complemented with either Flag-EXD2 WT or Flag-EXD2 nuclease dead (ND) mutant protein for G1 cells with 53BP1 OPT domains (mean ± SEM, n = 3 independent experiments, chi-square). (F) Quantification of HeLa WT, *EXD2*^−/−^, and *EXD2*^−/−^ cells complemented with either Flag-EXD2 WT or Flag-EXD2 nuclease dead (ND) mutant protein for cells showing MN (mean ± SEM, n = 3 independent experiments, chi-square).

Failure to fully complete DNA replication leads to cells entering mitosis with under-replicated DNA, causing chromosome breakage and inhibiting cells’ ability to segregate chromosomes (Burrell et al., 2013). This can be visualized as formation of anaphase bridges, 53BP1 OPT domains in daughter cells, and formation of micronuclei (MN) (Harrigan et al., 2011, Lukas et al., 2011). In support of EXD2’s role in promoting genome duplication, EXD2-deficiency correlates with high levels of anaphase bridges or 53BP1 OPT domains (Figures 4A and 4B). Additionally, the frequency of MN is also elevated by ∼2-fold in *EXD2*^−/−^ cells as compared to WT (Figure 4C). Importantly, only the WT protein, but not the nuclease-dead mutant, is capable of complementing these defects (Figures 4D–4F). Collectively, these data indicate that EXD2 promotes recovery of stressed forks thereby suppressing fork collapse and the generation of replication intermediates that may interfere with chromosomal segregation and drive genome instability.

### EXD2 Protects Replication Forks against Uncontrolled Degradation

**Figure 5:**
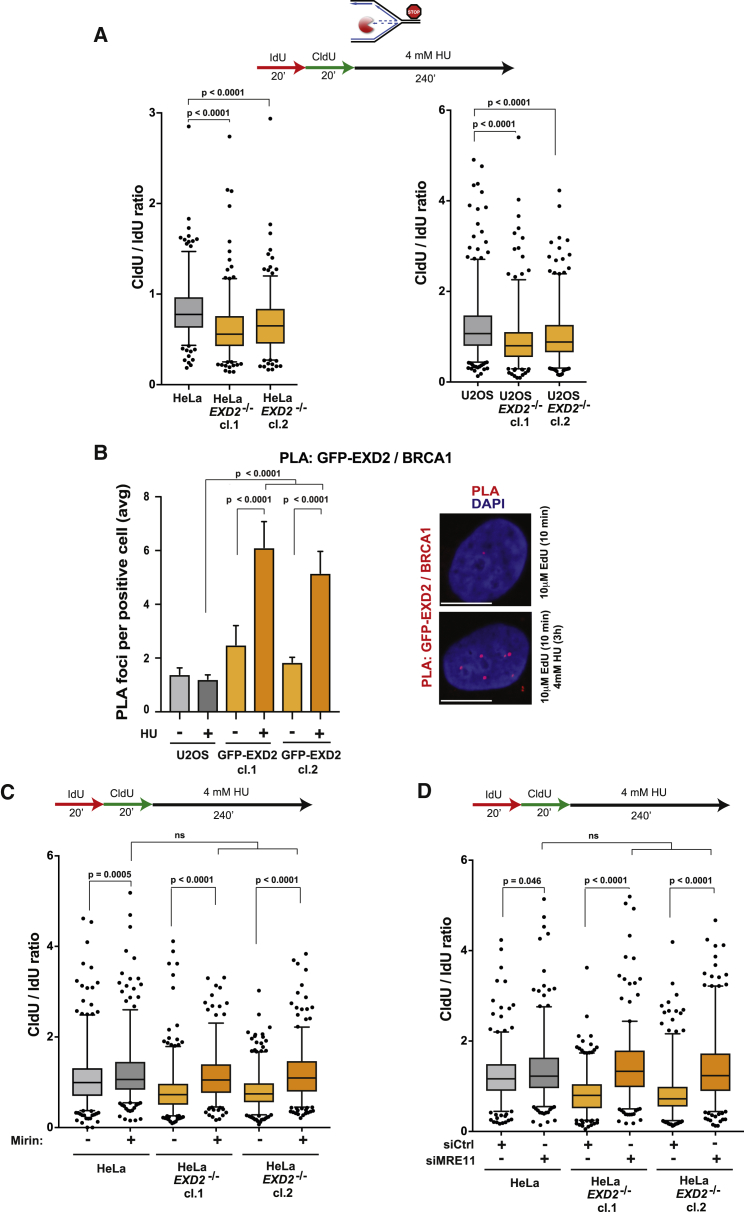
EXD2 Protects Replication Forks against Uncontrolled Degradation of Nascent DNA (A) Boxplot of CldU/IdU tract ratios of HeLa WT and *EXD2*^−/−^ cells (left panel) and U2OS WT and *EXD2*^−/−^ cells (right panel) (5–95 percentile, n ≥ 300 tracts pooled from 3 independent experiments, Mann-Whitney). (B) Quantification of U2OS cells or U2OS cells stably expressing GFP-EXD2 for GFP/BRCA1 PLA foci (mean ± SEM from 3 independent experiments, Mann-Whitney) and representative images. Scale bar, 10 μm. (C) Boxplot of CldU/IdU tract ratios of HeLa WT and *EXD2*^−/−^ cells untreated or pre-treated with MRE11 inhibitor Mirin (50 μM) (5–95 percentile, n ≥ 300 tracts pooled from 3 independent experiments, Mann-Whitney). (D) Boxplot of CldU/IdU tract ratios of HeLa WT and *EXD2*^−/−^ cells upon MRE11 knock-down (5–95 percentile, n ≥ 300 tracts pooled from 3 independent experiments, Mann-Whitney).

Recent work suggests that stressed replication forks undergo excessive nucleolytic degradation (Chaudhuri et al., 2016, Hashimoto et al., 2010, Karanja et al., 2014, Lachaud et al., 2016, Schlacher et al., 2011). This process drives genome instability and underpins the ability of cancer cells to become chemoresistant. Key players in this process are the BRCA1/2 proteins that inhibit MRE11 nuclease-dependent fork resection by regulating RAD51 loading onto stalled forks (Chaudhuri et al., 2016, Kolinjivadi et al., 2017b, Lemaçon et al., 2017, Mijic et al., 2017, Pasero and Vindigni, 2017). To determine the mechanism by which EXD2 protects stressed forks, we tested if EXD2 loss impacts on fork resection by employing a modified DNA fiber protocol as described recently (Chaudhuri et al., 2016). Briefly, WT and EXD2-deficient HeLa cells were incubated with IdU and CldU and then exposed to HU. Strikingly, EXD2-deficient cells also displayed significantly elevated fork resection compared to WT cells, reflecting degradation of newly replicated DNA after fork arrest (Figure 5A, left panel). Importantly, this seems to be a general, non-cell line restricted phenotype, as we observed a similar response in U2OS *EXD2*^−/−^ cells (Figure 5A, right panel). In line with EXD2’s role in protecting stalled forks, we also observed increased co-localization between EXD2 and the fork-protection factor BRCA1 upon replicative stress (Figure 5B).

Pathological degradation of unstable forks has been attributed to the deleterious activity of MRE11 (Chaudhuri et al., 2016, Kolinjivadi et al., 2017b, Mijic et al., 2017, Schlacher et al., 2011). Because EXD2 functionally interacts with MRE11 during homology-mediated repair (Broderick et al., 2016), we asked if MRE11 can drive fork processing upon loss of EXD2. To test this, we employed mirin, an MRE11 inhibitor shown to block fork resection (Schlacher et al., 2011, Taglialatela et al., 2017). Strikingly, treatment with mirin reversed the excess fork degradation seen in *EXD2*^−/−^ cells (Figure 5C). A similar result was achieved using small interfering RNA (siRNA) against MRE11 (Figures 5D and S3A). Because BRCA1/2 have been shown to prevent MRE11-dependent degradation of stressed forks by promoting RAD51 loading (Hashimoto et al., 2010, Schlacher et al., 2011), we considered the possibility that loss of EXD2 impairs recruitment or retention of RAD51 at nascent DNA. However, this does not seem to be the case, as we do not see any major change in the number of RAD51-dependent PLA foci at the sites of fork stalling in *EXD2*^−/−^ cells in contrast to BRCA1 knockdown (Figure S3B). Consistently, the level of BRCA1 loading at the vicinity of stalled forks is also similar between WT and *EXD2*^−/−^ cells (Figure S3C). Finally, and in contrast to BRCA1 deficiency (Taglialatela et al., 2017), we also did not observe an increased recruitment of MRE11 to stalled forks in *EXD2*^−/−^ cells (Figure S3D, note the increased level of MRE11 signal in cells depleted for BRCA1) (Taglialatela et al., 2017). Taken together, this data suggests a previously undescribed RAD51- and BRCA1/2-independent mechanism of fork protection underpinned by EXD2’s activity.

**Figure 6:**
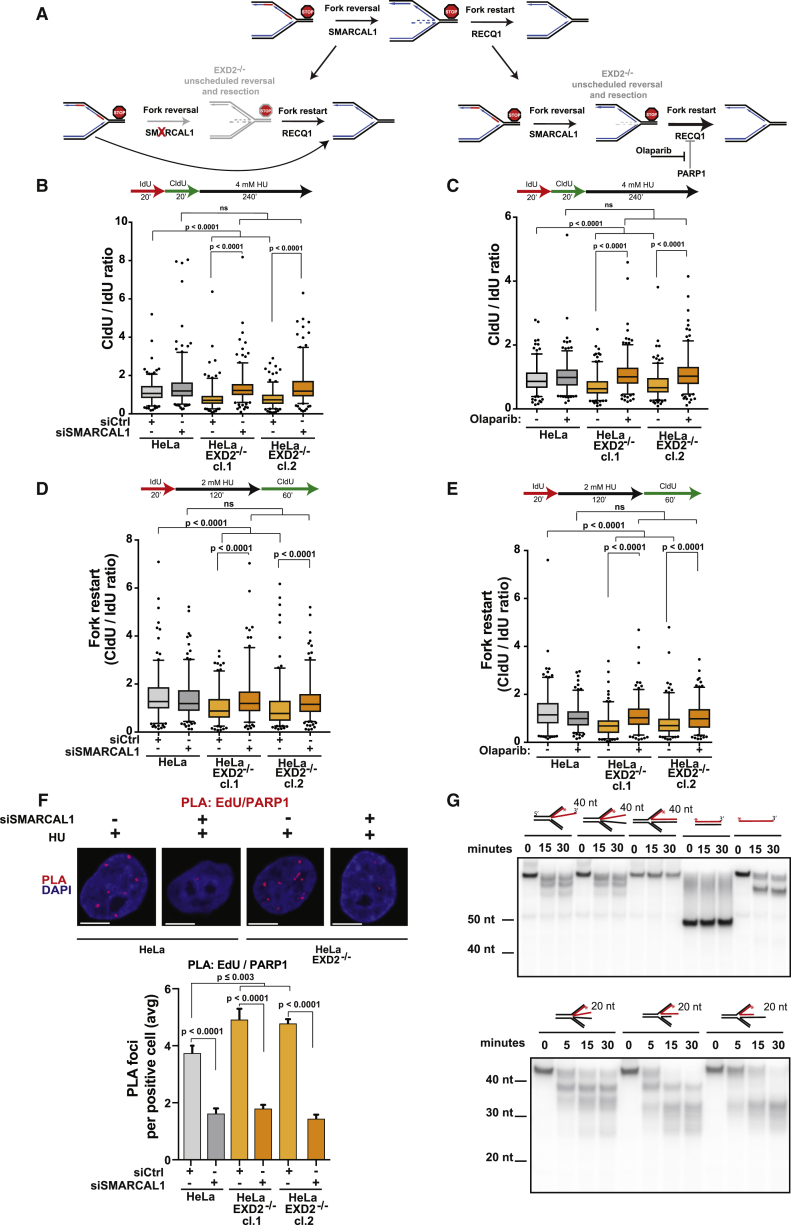
EXD2 Acts to Counteracts Replication Fork Regression (A) Schematic of the process of replication fork reversal and RECQ1-mediated restart of regressed forks (upper panel); upon loss of EXD2 reversed forks undergo pathological degradation. Knockdown of SMARCAL1 suppresses fork reversal and promotes fork restart (bottom left panel); RECQ1 is inhibited by parylation thus, PARP inhibition de-represses RECQ1 and increases its activity counteracting fork regression (bottom right panel). (B) Boxplot of CldU/IdU tract ratios of HeLa WT and *EXD2*^−/−^ cells upon SMARCAL1 knockdown (5–95 percentile, n ≥ 200 tracts pooled from 3 independent experiments, Mann-Whitney). (C) Boxplot of CldU/IdU tract ratios of HeLa WT and *EXD2*^−/−^ cells untreated or treated with 10 μM olaparib (5–95 percentile, n ≥ 200 tracts pooled from 2 independent experiments, Mann-Whitney). (D) Boxplot of CldU/IdU tract ratios of HeLa WT and *EXD2*^−/−^ cells upon SMARCAL1 knockdown (5–95 percentile, n ≥ 180 tracts pooled from 3 independent experiments, Mann-Whitney). (E) Boxplot of CldU/IdU tract ratios of HeLa WT and *EXD2*^−/−^ cells untreated or treated with 10 μM olaparib (5–95 percentile, n ≥ 160 tracts pooled from 3 independent experiments, Mann-Whitney). (F) Quantification of the average number of PLA foci per focus positive cell in HeLa and *EXD2*^−/−^ cells upon SMARCAL1 knockdown and representative images (mean ± SEM, n = 4 independent experiments, Mann-Whitney). Scale bar, 10 μm. (G) Phosphor imaging of 5′ radiolabeled indicated DNA substrates (labeled strand shown in red, length of the regressed arm indicated) incubated for indicated amounts of time with EXD2 WT protein.

Perturbation to replication fork progression in mammalian cells induces a high frequency of fork reversal (Zellweger et al., 2015). This process is controlled by the action of multiple fork remodelers, which promote efficient fork repair and restart. Unscheduled fork reversal, however, is toxic and results in pathological degradation of nascent DNA driving genome instability (Kolinjivadi et al., 2017b, Mijic et al., 2017, Neelsen and Lopes, 2015). We therefore wondered if fork degradation observed in *EXD2*^−/−^ cells could be due to its role in counteracting regression of stalled forks. We tested this hypothesis in several ways; first, we examined the impact of depleting the SMARCAL1 fork remodeler on fork degradation in cells lacking EXD2. Strikingly, SMARCAL1 knockdown suppressed resection of stressed forks in these cells (Figures 6A, 6B, and S3E). Second, fork reversal is counteracted by the action of the RECQ1 helicase, which is inhibited by PARP1 (Berti et al., 2013). We reasoned therefore, that inhibiting PARP1 should de-repress RECQ1 and increase its fork restoration activity (Berti et al., 2013, Margalef et al., 2018). In support of our hypothesis, treatment of EXD2-deficient cells with the PARP1 inhibitor olaparib abolished the resection phenotype of *EXD2*^−/−^ cells (Figures 6A and 6C). Unscheduled fork reversal coupled with fork degradation is a pathological process that inhibits replication fork restart. Therefore, if EXD2 acts to limit fork regression, limiting reversal in *EXD2*^−/−^ cells should improve the recovery of stalled forks. In support of this hypothesis, this is exactly what we observe. Downregulating SMARCAL1 or removing the PARP1-dependent inhibition of RECQ1 restores the ability of *EXD2*^−/−^ cells to resume DNA synthesis, and importantly, ameliorates the genome instability of these cells (Figures 6D, 6E, and S4A–S4C). Contrary to this, silencing of RECQ1 is epistatic with EXD2 deficiency suggesting that these two proteins function within the same mechanism to counteract fork regression (Figures S5A–S5D, and S5G [model]). However, because PARP1 plays multiple roles in protecting stalled forks, including the recruitment of MRE11 to promote HR-dependent fork restart (Bryant et al., 2009), we considered the possibility that inhibiting fork resection alone may be enough to rescue fork restart in *EXD2*^−/−^ cells. Importantly, this is not the case as treatment with mirin, which inhibits over-resection in *EXD2*^−/−^ cells, does not rescue the defective fork restart (Figure S5E). To further support this hypothesis, we analyzed PARP1 recruitment to nascent DNA using the PLA assay. We reasoned that because PARP1-catalyzed PARylation of RECQ1 at stalled replication forks regulates its activity (Berti et al., 2013), PARP1 accumulation on nascent DNA could serve as an indirect readout of fork regression (Margalef et al., 2018). In support of our hypothesis, we see a significant increase in the PLA signal for PARP1-EdU in cells lacking EXD2 as compared to control. Crucially, this is rescued by the knockdown of SMARCAL1 confirming that the PLA signal is derived from regressed forks (Figures 6F and S5F).

Reversed replication forks are predicted to arise via the translocation of the branch point of a stalled fork, followed by annealing of the two newly synthesized strands to create a 4-way junction. We hypothesized that EXD2-dependent nucleolytic processing of the regressed nascent strand(s) could counteract fork reversal. We therefore analyzed the ability of purified EXD2 to process structures mimicking reversed replication forks, including those with extruded nascent strands. Consistent with our hypothesis, EXD2 was able to degrade these substrates (Figures 6G and S6A–S6C). Collectively, these results support the conclusion that EXD2 nuclease-dependent fork processing counteracts fork regression and promotes restoration of active replication forks.

### EXD2 Depletion Is Synthetic Lethal with BRCA1/2

**Figure 7:**
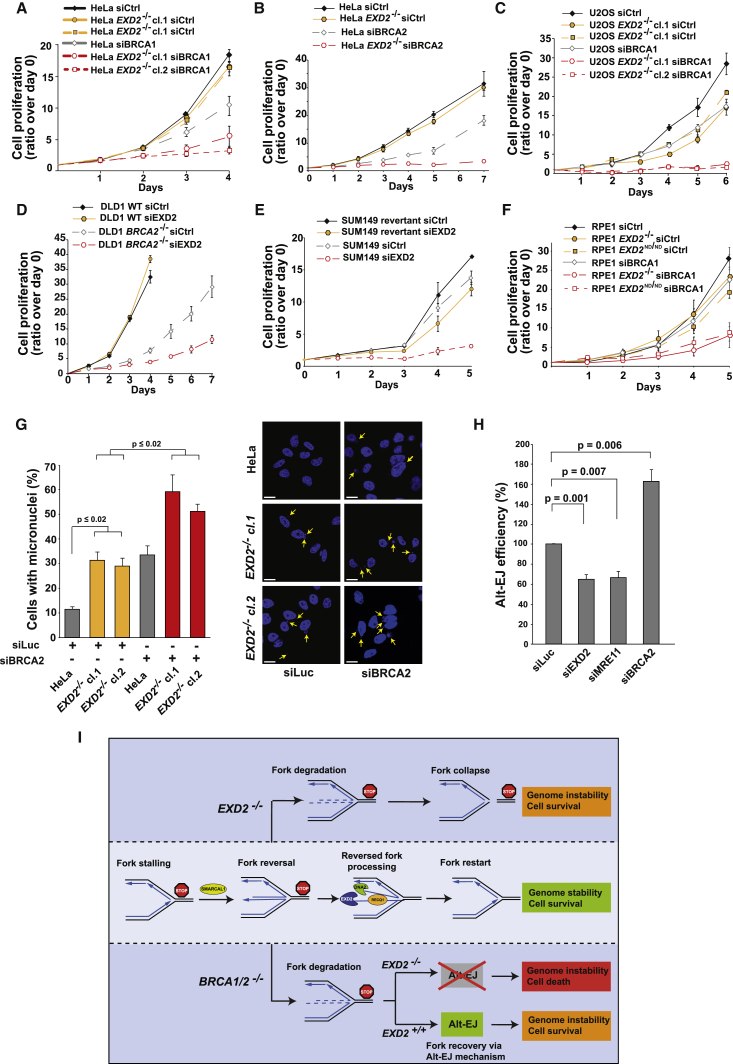
EXD2 Is Required for Survival of BRCA1/2-Deficient Cells (A) Proliferation of HeLa WT and *EXD2*^−/−^ cells upon BRCA1 knockdown (mean ± SEM, n = 3 independent experiments). (B) Proliferation of HeLa WT and *EXD2*^−/−^ cells upon BRCA2 knockdown (mean ± SEM, n = 3 independent experiments). (C) Proliferation of U2OS WT and *EXD2*^−/−^ cells upon BRCA1 knockdown (mean ± SEM, n = 4 independent experiments). (D) Proliferation of DLD1 WT and *BRCA2*^−/−^ cells upon EXD2 knockdown (mean ± SEM, n = 3 independent experiments). (E) Proliferation of SUM149 and SUM149 revertant cells upon EXD2 knockdown (mean ± SEM, n = 3 independent experiments). (F) Proliferation of RPE1 WT, *EXD2*^−/−^, and *EXD2*^ND/ND^ cells upon BRCA1 knockdown (mean ± SEM, n = 3 independent experiments). (G) Quantification of the HeLa WT and *EXD2*^−/−^ cells upon BRCA2 knockdown showing MN and representative images (mean ± SEM, n = 3 independent experiments). Scale bar, 20 μm. (H) Quantification of the relative alt-EJ efficiency upon EXD2, MRE11 or BRCA2 knockdown as indicated (mean ± SEM, n = 5 independent experiments, t test). (I) Proposed model for EXD2 function during genome duplication. Replicative stress leads to fork stalling. Stressed forks are protected by EXD2 activity in a pathway cooperating with RECQ1, allowing for efficient fork restart and timely accomplishment of DNA replication. Loss of EXD2 leads to extensive degradation of nascent DNA at stalled forks, compromising fork restart and ultimately adversely impacting genome stability. Combined deficiency in EXD2 and BRCA1/2 results in loss of fork protection and in the absence of functional HR also compromises cells’ ability to rescue collapsed forks by the backup Alt-EJ pathway.

Our analysis thus far suggested that EXD2 suppresses replicative stress via a mechanism that is distinct from the role of the BRCA1/2 genes in this process. We therefore considered the possibility that EXD2 is required to avert replication catastrophe in cells lacking BRCA1/2. With this in mind, we first analyzed the proliferative capabilities of both single and double mutants. Strikingly, combined depletion of EXD2 with BRCA1/2 in HeLa or U2OS cells leads to an almost complete inhibition of cell growth (Figures 7A–7C and S6D–S6F). Importantly, this phenotype could be recapitulated in BRCA1/2-deficient cancer cell lines (Dréan et al., 2017) (Figures 7D, 7E, S6G, and S6H) and is dependent on the nuclease activity of EXD2 (Figures 7F and S6I). To further our understanding of this synergistic interaction, we analyzed the frequency of micronuclei formation, as a readout of chromosomal instability. Consistent with the growth inhibition data, we noticed a marked increase in micronuclei formation in the double deficient mutants as compared to the singles (Figure 7G).

Surprisingly, we were unable to detect a significant increase in fork stalling or degradation in these double mutants (Figures S7A and S7B). Intriguingly, however, we noticed a dramatic deficiency of *EXD2*^−/−^ cells to generate chromosome end-to-end fusions (Figure S7C), which are dependent on the alternative end-joining pathway (Alt-EJ) (Mateos-Gomez et al., 2015). This pathway relies on limited resection of DSBs by the MRE11 nuclease to generate short stretches of homology used to bridge the break (Howard et al., 2015), and recent work has implicated Alt-EJ in survival of BRCA1/2-deficient tumors (Ceccaldi et al., 2015, Mateos-Gomez et al., 2015). Because EXD2 functionally interacts with the MRE11 nuclease (Broderick et al., 2016), we considered the possibility that the synthetic growth defect may reflect a lack of fork protection coupled with the loss of Alt-EJ to rescue unprotected forks. To test this, we depleted EXD2 in a U2OS cell line expressing the Alt-EJ reporter construct (Gunn et al., 2011), using BRCA2 and MRE11 knockdown as a positive and negative control, respectively. As previously reported, silencing of BRCA2 increased the level of Alt-EJ events whereas knockdown of MRE11 reduced its efficiency (Ceccaldi et al., 2015, Howard et al., 2015). Strikingly, we noticed that Alt-EJ was also significantly impaired in the absence of EXD2 (Figures 7H, S7D, and S7E). Collectively, these data suggest a role for EXD2 in promoting repair of chromosomal breaks in the absence of BRCA1/2 by an alternative end-joining pathway. Because Alt-EJ is essential for survival of BRCA1/2 mutants (Ceccaldi et al., 2015, Mateos-Gomez et al., 2015), we propose that combined depletion of BRCA1/2 and EXD2 results in an accumulation of broken replication forks, which cannot be rescued by the Alt-EJ backup mechanism driving mitotic catastrophe and cell death, even under normal growth conditions.

## Discussion

While addressing the key question of how cells maintain genome stability at stressed replication forks, we have determined that EXD2 is essential for an efficient response to replicative stress. We reveal that EXD2 is recruited to stalled forks with fast kinetics, similar to those observed for the MRE11 nuclease (Haince et al., 2008, Suhasini et al., 2013), and its loss compromises efficient replication fork progression. In line with this, *EXD2*^−/−^ cells display sensitivity to a range of agents that interfere with DNA replication, including clinically relevant anti-cancer drugs. In keeping with EXD2’s role in mitigating replicative stress, EXD2 mutant cells enter mitosis with under-replicated DNA, which leads to elevated levels of anaphase bridges, G1-associated 53BP1 OPT domains, and micronuclei, likely all of which contribute to the increased chromosome breakage observed in these cells. Given the significant increase in 53BP1 foci (a marker of DSBs) in *EXD2*^−/−^ cells during S-phase, we postulate that loss of EXD2 results in replication fork collapse.

Fork reversal has recently emerged as an important mechanism of cellular responses to replicative stress (Neelsen and Lopes, 2015). Uncontrolled fork reversal, however, can result in fork degradation, irreversible collapse, and genome instability (Kolinjivadi et al., 2017a, Pasero and Vindigni, 2017, Yeeles et al., 2013). Surprisingly, we discovered that EXD2 acts at stressed forks to suppress their uncontrolled regression, most likely by enzymatically processing stalled forks to restrict their conversion to reversed forks. Our conclusion is supported by the following observation; inhibition of fork regression by downregulation of SMARCAL1 or de-repressing PARP1-dependent inhibition of RECQ1 fork restoration activity rescues not only the excessive resection of stalled forks but also fork restart in *EXD2*^−/−^ cells. Consistently, examining the association of PARP1 with nascent DNA indicates a significant increase in fork reversal in the absence of EXD2, which is corrected by SMARCAL1 knockdown. Crucially, however, treatment with mirin, which inhibits fork resection in *EXD2*^−/−^ cells, does not rescue fork restart. One model to explain how EXD2 functions in counteracting fork reversal is that large single-stranded DNA (ssDNA) gaps formed behind the stalled fork (Kolinjivadi et al., 2017b), and/or the already regressed nascent leading strand reveals a 3′ DNA end that can be acted upon by EXD2. Subsequently, nucleolytic processing of such a substrate alone, or together with the resection of the opposite nascent strand, may promote re-annealing of the parental strands and fork backtracking (Pasero and Vindigni, 2017). A second model, which we favor, is that at the initial stage of fork regression, the nascent leading strand becomes dissociated (due to the spatial configuration of the fork, dissociated nascent strands may not be able to anneal with each other initially) or at the later stage, is actively displaced by RECQ1's helicase activity, and subsequently, EXD2-dependent resection of the unpaired strand would prevent fork reversal (i.e., formation of a 4-way junction). In turn, this would shift the balance toward restoration of an active fork. Indeed, EXD2 and RECQ1 deficiencies are epistatic and purified EXD2 is capable of degrading 3′ ssDNA within a substrate mimicking reversed forks with an extruded (unpaired) nascent strand. This could be an important mechanism restricting undesirable regression of a fork before the lagging strand is fully mature and/or upon transient dissociation of the leading strand during replisome staling. Because the two sister chromatids are physically linked at the replication fork, displacement of the leading nascent strand (i.e., due to a transient fork reversal) followed by RAD51 filament formation could initiate homology search, increasing ectopic HR-driven recombination, a considerable problem for highly repetitive genomes (Lambert et al., 2010, Mizuno et al., 2009, Mizuno et al., 2013). Of note, leading- and lagging-strand polymerases are not always coordinated, and ssDNA intermediates form even at active forks (Graham et al., 2017). Accordingly, processing of the dissociated nascent strands in bacteria and yeast has been shown to enable replication fork structures to be regenerated, limiting HR-dependent chromosomal rearrangements (Courcelle et al., 2003, Hu et al., 2012, Iraqui et al., 2012, Yeeles et al., 2013).

Interestingly, it has been proposed that efficient recovery of stalled forks in mammalian cells may require the action of an as yet unidentified 3′-5′ nuclease (Kolinjivadi et al., 2017a, Pasero and Vindigni, 2017). Therefore, it is tempting to speculate that EXD2, itself a 3′-5′nuclease, could be this missing factor. Consistent with this, our analysis shows that EXD2’s nuclease activity is essential for its role in promoting recovery of stressed forks and suppression of replication-associated genome instability.

### EXD2 Is Synthetic Lethal with BRCA1/2

Germline mutations in the BRCA1/BRCA2 genes account for up to 80% of familial breast and ovarian cancer cases (King et al., 2003, Prakash et al., 2015). In their absence, nascent DNA at the stalled fork is extensively degraded, likely contributing to BRCA1/2-associated genome instability and sensitivity to replication stress-inducing therapies (Ceccaldi et al., 2015, Chaudhuri et al., 2016). Our observation that loss of EXD2 induces excessive fork degradation that is not coupled to impaired RAD51 or BRCA1 association with stalled forks prompted us to analyze the genetic interaction between BRCA1/2 and EXD2. Surprisingly, we discovered that combined deficiency in these factors is incompatible with cell proliferation, even in the absence of any exogenous challenge. Therefore, unlike depletion of fork remodelers (Kolinjivadi et al., 2017b, Mijic et al., 2017, Taglialatela et al., 2017), loss of EXD2 aggravates genomic instability associated with BRCA1/2 deficiencies. Tumors deficient in one DNA repair pathway often rely on a compensatory mechanism to resolve the damage. Consistent with this notion, we show a role for EXD2 in promoting repair of DSBs via alternative end-joining (Alt-EJ). Thus, in the absence of both EXD2 and BRCA1/2 proteins, unprotected replication forks collapse and cannot be rescued by either canonical HR or the Alt-EJ backup mechanism, ultimately compromising cell viability.

In conclusion, we show that EXD2 promotes efficient genome duplication by counteracting regression of stalled forks (Figures 7I and S5G). Since replicative stress is one of the major drivers of tumorigenesis and aging (Flach et al., 2014, Gaillard et al., 2015, Halazonetis et al., 2008), this work provides an important insight into mechanisms protecting cells against acquisition of DNA damage. In addition, our results offer a potential therapeutic target for tumors with BRCA1/2 deficiency. In the longer term, it would be worthy to assess the penetrance of this phenotype in other HR-deficient cancers.

## STAR★Methods

### Key Resources Table

**Table:** 

REAGENT or RESOURCE	SOURCE	IDENTIFIER
**Antibodies**		

α-Tubulin	Sigma	T5168; RRID: AB_477579
BRCA1	Millipore	OP-92; RRID: AB_2750876
BRCA2	Millipore	OP-95; RRID: AB_2067762
EXD2	Sigma-Aldrich	HPA005848; RRID: AB_1078768
MCM2	Abcam	ab4461; RRID: AB_304470
MRE11	Abcam	ab214; RRID: AB_302859
PCNA	Santa-Cruz	PC-10; RRID: AB_628110
RECQ1	Santa-Cruz	SC-166388; RRID: AB_2178425
SMARCAL1	Santa-Cruz	Sc-376377; RRID: AB_10987841
53BP1	Millipore	MAB3802; RRID: AB_2206767
Cyclin A	Santa-Cruz	sc751; RRID: AB_631329
Biotin	Bethyl Laboratories,	A150-109A; RRID: AB_67327
Biotin	Jackson Immunoresearch	200-002-211; RRID: AB_2339006
BRCA1	Santa-Cruz	sc6954; RRID: AB_626761
Donkey anti-Mouse IgG (H+L) Secondary Antibody, Alexa Fluor 555	Thermo Fisher Scientific	A31570; RRID: AB_2536180
Donkey Anti-Rabbit IgG (H+L) Antibody, Alexa Fluor 488 Conjugated	Thermo Fisher Scientific	A21206; RRID: AB_141708
Anti-BrdU antibody; Rat monoclonal [BU1/75 (ICR1)]	Abcam	ab6326; RRID: AB_305426
Purified Mouse Anti-BrdU Clone B44	BD Biosciences	347580; RRID: AB_400326
Sheep anti-mouse Cy3 antibody	Sigma-Aldrich	C2181; RRID: AB_258785
Goat anti-Rat IgG (H+L) Cross-Adsorbed Secondary Antibody, Alexa Fluor 488	Thermo Fisher Scientific	A11006; RRID: AB_2534074

**Chemicals, Peptides, and Recombinant Proteins**		

PreScission Protease	GEHealthcare	27084301
Hydroxyurea	Sigma-Aldrich	H8627
Gemcitabine hydrochloride	Sigma-Aldrich	G6423-10MG
5-Iodo-2′-deoxyuridine	Sigma-Aldrich	I7125
5-Chloro-2′-deoxyuridine	MP Biomedicals	105478
Mitomycine C	Sigma-Aldrich	M4287
Cis-diamineplatinum dichloride	Sigma-Aldrich	P4394
Mirin	Sigma- Aldrich	M9948
Olaparib AZD2281	Seleck Chem	S1060
Lipofectamine 2000	Thermo Fisher Scientific	11668019
HiPerfect	QIAGEN	301707
Vectashield	Vector Lab	H-1000
Vectashield with DAPI	Vector Lab	H-1200
Immobilon Western Chemiluminescent HRP substrate	Millipore	WBKLS0500

**Critical Commercial Assays**		

Duolink^®^*In Situ* Red Starter Kit Mouse/Rabbit	Sigma-Aldrich	DUO92101-1KT
Invitrogen Molecular Probes Click-iT EdU Imaging Kit with Alexa Fluor 488, 594, and 647 Azides	Fischer Scientific	13435356
Biotin-dPEG_7_-azide	Quanta Biodesign/ Stratech	10825-QUA
EdU (5-ethynyl-2′-deoxyuridine)	Thermo Fisher Scientific	E10187

**Deposited Data**		

Raw image files	This study, Mendeley	https://doi.org/10.17632/gbc75sg6c7.1

**Experimental Models: Cell Lines**		

HeLa	From Fumiko Esashi Laboratory	N/A
HeLa S3	From Ian D. Hickson Laboratory	N/A
U2OS	From Fumiko Esashi Laboratory	N/A
RPE-1	From Andrew Blackford Laboratory	N/A
HEK293FT	From Grant Stewart Laboratory	N/A
SUM149	From Chris Lord Laboratory	Dréan et al., 2017
SUM149 revertant	From Chris Lord Laboratory	Dréan et al., 2017
DLD1	From Chris Lord Laboratory	Dréan et al., 2017
DLD1 BRCA2 ^−/−^	From Chris Lord Laboratory	Dréan et al., 2017
HeLa EXD2 ^−/−^	Wojciech Niedzwiedz Laboratory,	Broderick et al., 2016
U2OS EXD2 ^−/−^	This study	N/A
RPE1 EXD2 ^−/−^	This study	N/A
RPE1 EXD2 ND/ND	This study	N/A
HeLa EXD2 ^−/−^ + Flag-HA-EXD2 WT	This study	N/A
HeLa EXD2 ^−/−^ + Flag-HA-EXD2 ND	This study	N/A
U2OS + GFP-EXD2	This study	N/A
U2OS + EXD2-GFP	This study	N/A

**Oligonucleotides**		

gRNA1; GTCTAATTCACTTCTAAGCAA	This study	N/A
gRNA2; GACTTGGAATTGACTGTGAGT	This study	N/A
ssODN_ND; AGGAGGCAGAGTGGGATCAAAT CGAGCCCTTGCTTAGATCTGAATTAGAAGATT TTCCAGTACTTGGTATCGCTTGTGCGTGGGTA AGTTAAAAAGCAAAAGTTAAAAAA	This study	N/A
siBRCA1- ACCAUACAGCUUCAUAAAUAA	This study	N/A
siBRCA2 ON-TARGETplus SMART pool,	Dharmacon	L-003462-00-0005
siEXD2 – CAGAGGACCAGGUAAUUUA	Dharmacon	Broderick et al., 2016
siEXD2-2 (ON-TARGETplus EXD2 siRNA)	Dharmacon	L-020899-02-0005
siEXD2 (3′ UTR) GAACAAGGAGUCAAAUUUA	Dharmacon	Broderick et al., 2016
siSMARCAL1 ON-TARGETplus SMARTpool	Dharmacon	L-013058-00-0005)
siMRE11 GGAGGUACGUCGUUUCAGA	Dharmacon	Broderick et al., 2016
siRECQ1 ON-TARGETplus SMARTpool	Dharmacon	L-013597-00-0005
siLuciferase CGTACGCGGAATACTTCGA	Dharmacon	Broderick et al., 2016
ON-TARGETplus Non-targeting Pool	Dharmacon	D-00180-10-20

**Recombinant DNA**		

pGEX6-His-EXD2 K76-V564	This study	N/A
EXD2-peGFPN2	This study	N/A
EXD2-pHAGE-N-Flag–HA	Broderick et al., 2016	N/A
EXD2-pDEST-peGFP	This Study	N/A
pCMV-I-Sce1	a kind gift from Dr. V. Macaulay	N/A
pmCherry-C1	Clontech	N/A

**Software and Algorithms**		

ImageJ	NIH	https://imagej.nih.gov
GraphPad Prism v7.00	GraphPad Prism version 7.00 GraphPad Software, La Jolla California USA	https://graphpad.com

### Lead Contact and Materials Availability

Further information and requests for resources and reagents should be directed to the Lead Contact, Wojciech Niedzwiedz (wojciech.niedzwiedz@icr.ac.uk).

### Experimental Model and Subject Details

HeLa and U2OS cells were a generous gift from Dr F. Esashi and were cultured in Dulbecco’s modified Eagle’s medium (DMEM) supplemented with 10% fetal bovine serum (FBS) and standard antibiotics. HEK293FT cells and HeLa S3 cells were a generous gift from Dr G. Stewart and Prof. I.D. Hickson, respectively, and were cultured in DEMEM supplemented with 10% FBS and standard antibiotics. U2OS cells stably expressing GFP-CtIP were a generous gift from Prof. S.P. Jackson and were cultured in media supplemented with 500 μg ml^-1^ G-418. U2OS EJ2-GFP cells were a kind gift of Prof. J. Stark. DLD1 WT and DLD1 BRCA2^−/−^ cell lines were a kind gift from Prof. C. Lord and were maintained in RPMI media supplemented with 10% fetal bovine serum, 2mM L-Glutamine and standard antibiotics. SUM149 and SUM149 revertant cell lines were a kind gift of Prof. C. Lord and were cultured in Ham’s F-12 medium supplemented with 10% fetal bovine serum, 5 μg/mL insulin, and 1 μg/mL hydrocortisone. U2OS cells stably expressing FLAG-HA EXD2 (generated previously) were cultured in DMEM supplemented with 0.5 μg ml^-1^ Puromycin (GIBCO).

### Method Details

#### Cell Lines Generation

Cell lines stably expressing GFP EXD2 WT fusion proteins were generated by transfection of U2OS cells with this plasmid construct followed by clonal selection of cells grown in media containing 500 μg ml^-1^ G418 (Life Technologies). HeLa *EXD2*^−/−^ cell lines stably expressing FLAG-HA EXD2 WT or D108A/E110A fusion proteins were generated by transfection of these cells with plasmid construct followed by clonal selection of cells grown in media containing 0.25 μg ml^-1^ Puromycin (GIBCO).

U2OS and RPE1 *EXD2*^*−/−*^ cells were generated as previously described (Broderick et al., 2016). RPE1 *EXD2*^*ND/ND*^ (D108A/E110A) were generated using following gRNAs GTCTAATTCACTTCTAAGCAA and GACTTGGAATTGACTGTGAGT cloned into pAIO-NK vector (a kind gift from Dr. A. Blackford) and ssODN AGGAGGCAGAGTGGGATCAAATCGAGCCCTTGCTTAGATCTGAATTAGAAGATTTTCCAGTACTTGGTATCGCTTGTGCGTGGGTAAGTTAAAAAGCAAAAGTTAAAAAA.

#### Plasmids and Cloning

Plasmid constructs employed to generate cell lines stably expressing FLAG EXD2 WT and FLAG EXD2 D108A/E110A were as previously described (Broderick et al., 2016). Plasmids expressing GFP-EXD2 were generated by cloning *EXD2* into the pDEST-peGFP (a generous gift from Prof C. Green) or the peGFPN2 plasmid (Clontech). Plasmids were transfected into human cells using Lipofectamine 2000 (Life Technologies), according to the manufacturer’s instructions. GST-His-EXD2 K76-V564 construct was obtained by cloning His-EXD2 K76-564 (Broderick et al., 2016) into the pGEX-6P1 vector. The pCMV-I-Sce1 plasmid was a kind gift from Dr V. Macaulay. pmCherry-C1 was obtained from Clontech.

#### Immunoblotting

Cell lysis was carried out in urea buffer (9 M urea, 50 mM Tris HCL, pH 7.3, 150 mM β-mercaptoethanol) followed by sonication using a soniprep 150 (MSE) probe sonicator. In some instances, cells were lysed in SDS loading buffer (2% SDS, 10% (v/v) glycerol, 2% 2-Mercaptoethanol and 62.5 mM Tris-HCl, pH 6.8) followed by boiling for 10 min. Samples were resolved by SDS-PAGE and transferred to PVDF or nitrocellulose. Protein concentrations were determined by Bradford assay by spectrophotometry using a NanoDrop 2000 device (Thermo Scientific). Immunoblots were carried out using the indicated antibodies: α-Tubulin (Sigma, B-5-1-2; T5168, 1:100,000), BRCA1 (Millipore, OP-92, 1:1000), BRCA2 (Millipore, OP-95, 1:1000), EXD2 (Sigma, HPA005848, 1:1000), MCM2 (Abcam, ab4461, 1:10,000), MRE11 (Abcam, ab214, 1:1000), PCNA (Santa-Cruz, PC-10, 1:500), RECQ1 (Santa Cruz, sc-166388,1:1000) and SMARCAL1 (Santa Cruz, sc-376377 1:1000).

#### Cell Survival and Proliferation Assays

Alamar Blue survival assays were performed in accordance with the manufacturer’s recommendations (Life Technologies). Briefly, 500 cells per well in 96-well plates were untreated or treated with indicated doses of camptothecin or ionising radiation and incubated for 7 days. Alamar blue reagent (Life Technologies) was added to each well and fluorometric measurements taken after 2h incubation at 37°C. For proliferation assays cells were seeded at 500 cells per well and Alamar blue reagent added and measurements taken each day as indicated.

#### RNAi treatment

siRNAs employed were as follows, siBRCA1- ACCAUACAGCUUCAUAAAUAA, siBRCA2 (ON-TARGETplus SMART pool L-003462-00-0005, Dharmacon.), siEXD2 – CAGAGGACCAGGUAAUUUA, siMRE11 – GGAGGUACGUCGUUUCAGA, siRECQ1 (ON-TARGETplus SMARTpool L-013597-00-0005) siSMARCAL1 (ON-TARGETplus SMARTpool L-013058-00-0005). ON-TARGETplus Non-targeting Pool (D-00180-10-20, Dharmacon), or siRNA targeting luciferase - CGTACGCGGAATACTTCGA were used as control siRNAs where appropriate. Oligonucleotides were transfected using HiPerfect reagent (QIAGEN), according to the manufacturer’s protocol.

### Immunofluorescence microscopy

For visualization of 53BP1 foci and OPT domains in Cyclin A-negative and -positive cells, respectively, cells were fixed with 4% paraformaldehyde in PBS for 10 min at room temperature, washed twice in PBS and permeabilised with 0.2% Triton Triton X-100 in PBS for 10 min at room temperature. Coverslips were washed 3 x in PBS and blocked in 10% FBS in PBS for 30 min before incubation with primary antibodies in 0.1% FBS in PBS for 1h at room temperature, washed 4 × 5 min in PBS temperature followed by incubation with secondary antibodies for 45 min. Slides were then washed 4 × 5 min in PBS and mounted with Vectashield mounting medium (Vector Laboratories) with DAPI. Antibodies employed for immunofluorescence were as follows: 53BP1 (MAB3802, Millipore, 1:1000), Cyclin A (sc751, Santa Cruz, 1:100).

For Phalloidin staining, cells were fixed and permeabilised as above and incubated with PBS containing Alexa Fluor 647- Phalloidin (Thermo Fisher A22287, 1:50) before being mounted using Vectashield with DAPI.

For analysis of anaphase bridges cells were analyzed using a protocol adapted from Broderick et al. (2015). Briefly, cells were collected by mitotic shakeoff and spun onto poly-L-Lysine coated slides at 1000 x g for 3 min. Mitotic cells were then fixed using 4% PFA in PBS for 10 min at room temperature and mounted with Vectashield containing DAPI. Images were acquired using a Zeiss LSM 710 laser scanning confocal microscope with Zen software using a 63x objective. Image analysis was carried out with FIJI (ImageJ) software.

#### EdU labeling of nascent DNA and Proximity Ligation Assay

The association of proteins to newly synthesized DNA using EdU labeling and the Proximity Ligation Assay was carried out as previously described (Taglialatela et al., 2017). Briefly, U2OS cells or U2OS cells stably expressing GFP or FLAG-HA EXD2 were grown on coverslips before being labeled with 10 μM EdU for 10 min followed in some cases by treatment with 4mM hydroxyurea for various amounts of time as indicated.

Cells were then permeabilized using 0.5% Triton in PBS for 10 min at 4°C, washed twice in PBS and fixed at with 3% formaldehyde, 2% sucrose in PBS for 10 min at room temperature. Post-fixation, cells were washed twice PBS and incubated with blocking solution (3% BSA in PBS) for 30 min. Slides were washed twice with PBS before conjugation of Biotin Azide to the newly incorporated EdU by click chemistry using the Click-iT reaction. The Click-iT reaction was carried using a Cick-iT assay kit (Thermo Fisher) according to the manufacturer’s instructions using 20 μM biotin-azide for 30 min. Coverslips were washed twice with PBS before incubation with the indicated primary antibodies for 1h at room temperature in 1% BSA/0.1% saponin in PBS.

Following primary antibody incubation coverslips were washed twice in PBS and then the proximity ligation assay was carried out using the Duolink *In Situ* Red Starter kit (Sigma Aldrich) according to the manufacturer’s instructions. Coverslips were mounted using Vectashield containing DAPI. For the PLA assay between GFP-EXD2 and BRCA1, cells were treated with HU for the indicated time before fixation in methanol at −20°C for 20 min followed by 3 washes with PBS. Blocking, primary antibody incubation and the PLA assay were then carried out as described above. Antibodies employed for the PLA assay were as follows: Biotin (Bethyl Laboratories, A150-109A, 1:3000), Biotin (Jackson Immunoresearch, 200-002-211, 1:1000), BRCA1 (Santa Cruz, sc6954, 1:500), FLAG (Sigma, M2, 1:500), (GFP (Abcam, ab290, 1:500), GFP (Roche, 11 814 460 001, 1:500), MRE11 (Abcam, ab214, 1:100), PARP1 (Santa Cruz, sc-5364, 1:500), Rad51 (Calbiochem, PC130, 1:500). Images were acquired using a Zeiss LSM 710 laser scanning confocal microscope with Zen software using a 63x objective. Image analysis was carried out with FIJI (ImageJ) software.

#### iPOND

Logarithmically growing HeLa S3 cells (1 × 10^6^ per ml) or HEK293FT cells were incubated with 10 μM EdU for 10 minutes. Following EdU labeling, cells were fixed in 1% formaldehyde, quenched by adding glycine to a final concentration of 0.125 M and washed three times in PBS. Collected cell pellets were frozen at −80°C and cells were permeabilized by resuspending 1.0–1.5 × 10^7^ cells per ml in ice cold 0.25% Triton X-100 in PBS and incubating for 30 minutes. Before the Click reaction, samples were washed once in PBS containing 0.5% BSA and once in PBS. Cells were incubated for 1 hour at room temperature in Click reaction buffer containing 10 μM azide-PEG(3+3)-S-S-biotin conjugate (Click ChemistryTools, cat. no AZ112-25), 10 mM sodium ascorbate, and 1.5 mM copper (II) sulfate (CuSO4) in PBS. The ‘no Click’ reaction contained DMSO instead of biotin-azide. Following the Click reaction, cells were washed once in PBS containing 0.5% BSA and once in PBS. Cells were resuspended in lysis buffer (50 mM Tris–HCl pH 8.0, 1% SDS) containing protease inhibitor cocktail (Sigma) and sonicated with a Diagenode Bioruptor® Plus for 40 cycles (30 s on/30 s off). Samples were centrifuged at 14,500 r*cf.* at 4°C for 30 minutes and the supernatant was diluted 1:3 with TNT buffer (50 mM Tris pH 7.5, 200 mM NaCl and 0.3% Triton X-100) containing protease inhibitors. An aliquot was taken as an input sample. Streptavidin–agarose beads (Novagen) were washed three times in TNT buffer containing protease inhibitor cocktail. Two hundred microliters of bead slurry was used per 1x 10^8^ cells. The streptavidin–agarose beads were resuspended 1:1 in TNT buffer containing protease inhibitors and added to the samples, which were then incubated at 4°C for 16 hours in the dark. Following binding, the beads were then washed two times with 1 mL TNT buffer, two times with TNT buffer containing 1M NaCl, two times with TNT buffer and protein–DNA complexes were eluted by incubating with 5 mM DTT in TNT buffer. Cross-links were reversed by incubating samples in SDS sample buffer at 95°C for 20 minutes. Proteins were resolved on SDS-PAGE and detected by immunoblotting using specific antibodies.

#### DNA fiber analysis

DNA fiber assay was performed as described previously with some modifications (Schwab et al., 2015). In brief, exponentially growing cells were first incubated with 25 μM iododeoxyuridine (IdU) and then with 125 μM chlorodeoxyuridine (CldU) for the indicated times. Fiber spreads were prepared from 0.5 x10^6^ cells/ml. Slides were stained as described previously . A confocal microscope (LSM 510 Meta or LSM 710 Meta; Carl Zeiss) equipped with Plan-Apochromat 63 × /1.4 oil DIC objective was used to collect fiber images from randomly selected fields at RT using ZEN 2009 software (Carl Zeiss). Analysis was performed using the ImageJ software package (National Institutes of Health). A minimum of 100 fibers or 20 sister fork pairs per experiment from at least three independent experiments was scored. Mann-Whitney test was used to determine statistical significance. On boxplots whiskers indicate 5-95 percentile.

#### Protein Purification

GST-His- EXD2 K76-V564 was purified as described previously with some modifications. Briefly, GST protein expression was induced with 0.1 mM IPTG (isopropyl-β-d-thiogalactopyranoside) (Sigma-Aldrich) at 16°C for 18 hours. Bacteria were harvested by centrifugation and resuspended in lysis buffer containing 50 mM phosphate pH 8.0, 300mM NaCl, 1 mM DTT, 1% Triton X-100, 10 mM imidazol and PMSF. Lysates were sonicated and cleared by centrifugation. Supernatants were incubated with Ni resin (QIAGEN) for 2 h with rotation at 4°C. Beads were washed with lysis buffer containing 20 mM imidazol, and eluted with lysis buffer containing 300 mM imidazole. Eluates were then incubated with Glutathione HiCap Matrix (QIAGEN) for 2 h with rotation at 4°C. Beads were washed with buffer containing increasing concentration of NaCl, elution buffer (50 mM Tris-HCl pH 7.0, 150 mM NaCl, 1 mM EDTA, 1 mM DTT, 0.2% Triton X-100) and resuspended in elution buffer supplemented with PreScission Protease (50 units/ml) (GE Healthcare) and incubated for 18 h with rotation at 4°C. Eluates were dialysed to buffer containing 20 mM HEPES-KOH pH7.2, 100 mM NaCl, 1 mM DTT, 10% glycerol, aliquoted and stored at −80°C.

#### *In vitro* nuclease assay

Sequences of DNA oligos used are listed in Table S1. To generate 5′ end labeled substrates, the indicated ssDNA oligo was labeled using [γ-^32^P] dATP and PNK enzyme (New England Biolabs). To obtain fork substrates, ssDNA oligos (as indicated in Table S1) were mixed in an equimolar ratio and annealed by heating at 100°C for 5 min followed by gradual cooling to room temperature.

Exonuclease assays were performed as described. Briefly, reactions were carried out in a buffer containing 20 mM HEPES-KOH, pH 7.5, 50 mM KCl, 0.5 mM DTT, 10 mM MnCl_2_, 0.05% Triton-X, 0.1 mg ml^-1^ BSA, 5% glycerol, and EXD2 protein (25 nM unless stated otherwise) and initiated by adding substrate (3nM unless stated otherwise) and incubated at 37°C for the indicated amounts of time. Reactions were stopped by addition of EDTA to a final concentration of 20 mM and 1/5 volume of formamide. The samples were resolved on denaturing 15% or 20% polyacrylamide TBE-Urea gels. Gels were fixed, dried and visualized using a Typhoon FLA 9500 instrument (GE Healthcare).

#### Recruitment of GFP-tagged proteins to laser localized DNA damage

Cells were grown on glass bottomed culture dishes (MatTek^™^, 35 mm). Plates were placed in an environmental chamber maintained at 37°C, 5% CO_2_, 80% humidity, mounted on the stage of a Nikon TE2000 spinning disk microscope. Cells were visualized with a Plan Fluor × 60/1.25 numerical aperture oil objective. The microscope was equipped with an SRS NL100 nitrogen laser-pumped dye laser (Photonics Instruments, St. Charles, IL). To introduce DNA damage a defined region of interest (ROI, 4 × 20 pixels, 0.16 μm/pixel) in an individual nucleus was exposed to the laser firing 3-ns pulses at 365 nm with a repetition rate of 10 Hz. The laser was controlled by Volocity-5 software (Improvision; PerkinElmer Life Sciences). The beam was oriented by galvanometer-driven displacers and fired randomly throughout the region until the entire region was exposed. Images were taken before targeting and at defined intervals after targeting to detect the GFP-tagged protein recruitment to the targeted stripe. Volocity Software was used to quantify the intensity of the GFP signal at the damage site and at an equivalent area in a non-targeted region of the nucleus (background). Graphs show [GFP Intensity at the stripe/ GFP intensity at non-targeted stripe in the same nucleus] as a function of time in arbitrary units. The average of at least 10 cells was graphed for each experiment. Error bars represent the standard deviation.

#### Chromosomal aberrations analysis

Sub-confluent cultures of HeLa WT and the of HeLa *EXD2*^−/−^ cl.1 and cl.2 cells, grown in DMEM with 10% FBS (GIBCO-BRL) plus antibiotics, at 37°C, were exposed to DMF (Sigma), 20 μM Cisplatin (Sigma) diluted in DMF for 16 h or 4 Gy X-ray irradiation. Two hours before harvest, the cells were exposed to colcemid (0.1μg/ml) (GIBCO). Cells were harvested by trypsinization (GIBCO), re-suspended in culture medium and then spun down (10 minutes at 1000 rpm). Supernatant was removed, and then 0.075 M KCl (Sigma) at room temperature was added drop by drop. For hypotonic treatment, cell suspensions were incubated for 20 minutes at room temperature, and then 1 mL of fixative 3:1 methanol (Applichem GmbH, Darmstadt, Germany)-acetic acid (Merck, Darmstadt, Germany) was added. Cells were re-centrifuged for 10 minutes at 1000 rpm, supernatant was removed, fixative was added, and then re-centrifuged in fixative for another two times. Finally, chromosome preparations were dropped onto wet microscope slides and left to air-dry. For inverted DAPI chromosome banding staining, slides were mounted with 0.1μg/ml DAPI in Vectashield antifade medium (Vector Laboratories, Burlingame, CA). Images of chromosome spreads were captured using a x63 magnification lens on a fluorescent Axio-Imager Z1, Zeiss microscope, equipped with a MetaSystems charge-coupled device camera and the MetaSystems Isis software. Chromosome lesions were recorded as breaks per chromosome number, per metaphase, in 75 metaphase spreads per condition, pooled from 3 independent experiments.

#### Alt-EJ GFP reporter assay

48 hours after siRNA transfection, U2OS EJ2-GFP cells (Gunn et al., 2011) were transfected using Amaxa nucleofection with an I-SceI expression vector (pCMV-I-SceI) or a vector expressing mCherry fluorescent protein (pmCherry-C1). 72 hours after I-SceI transfection cells were harvested and analyzed by flow cytometry (BD LSR II). 2x10^4^ cells were analyzed per experimental condition. Number of GFP-positive cells per 1000 mCherry-positive cells was determined using BD FACS DIVA software. The data were then related in each experiment to siControl treated sample set as 1. Statistical significance was determined with the Student’s t test.

### Quantification and Statistical Analysis

Statistical calculations were done using GraphPad Prism 7 (GraphPad Software Inc.). Unpaired Student’s t test, Chi square test or Mann Whitney test were used to determine statistical significance as indicated in the Figure Legends. Sample sizes are indicated in the Figure Legends.

## Supplemental Information

Document S1. Figures S1–S7 and Table S1

Document S2. Article plus Supplemental Information
