# Supplementary material for: EXD2 Protects Stressed Replication Forks and Is Required for Cell Viability in the Absence of BRCA1/2
Source: Mol Cell. 2019 Aug 8;75(3):605–619.e6. doi: 10.1016/j.molcel.2019.05.026 (PMC6695479; doi:10.1016/j.molcel.2019.05.026)
Supplement: Document S1. Figures S1–S7 and Table S1 [file mmc1.pdf]

Molecular Cell, Volume *75*

Supplemental Information

EXD2 Protects Stressed Replication Forks and Is Required for Cell Viability in the Absence of BRCA1/2

Jadwiga Nieminuszczy, Ronan Broderick, Marina A. Bellani, Elizabeth Smethurst, Rebekka A. Schwab, Veronica Cherdyntseva, Theodora Evmorfopoulou, Yea-Lih Lin, Michal Minczuk, Philippe Pasero, Sarantis Gagos, Michael M. Seidman, and Wojciech Niedzwiedz

SUPPLEMENTARY FIGURE LEGEND

Figure S1 – related to Figure 1

A) Table showing proteins identified in iPOND/MS analysis.

B) Western blot analysis of iPOND samples from A, confirming association of EXD2 with the newly replicated DNA.

C) Representative images of PLA anti-biotin/-; and anti-biotin/ anti-biotin negative and positive controls for this assay, respectively. Scale bar = 20μm.

D) Western blot confirming stable expression of GFP-EXD2 protein in U2OS cells. Two independent clones are shown. MCM2 serves as a loading control.

E) Percentage of cells with Flag/Biotin PLA foci (mean, +/- SEM, n=3 independent experiments). U2OS control cells and U2OS cells expressing Flag-EXD2 were left untreated or were treated with 4 mM HU for 3h as indicated; (red dots indicate close proximity between analysed targets), DAPI was used as a nuclear counterstain. Statistical significance was determined using the Student's t-test. Scale bar = 10μm.

F) Percentage of cells with GFP/Biotin PLA foci (mean, +/- SEM n=3 independent experiments). U2OS control cells and U2OS cells expressing C-terminally-tagged GFP-EXD2 (EXD2-GFP (C-term)) were left untreated or were treated with 4 mM HU for 3h as indicated; (red dots indicate close proximity between analysed targets), DAPI was used as a nuclear counterstain. Statistical significance was determined using the Student's t-test. Expression of EXD2-GFP is confirmed by western blotting using antibodies against EXD2 and PCNA which acts as a loading control. Scale bar = 10μm.

Figure S2 – related to Figures 1-3

A and B) Representative images of GFP-EXD2 and MRE11 recruitment to nascent DNA upon HU treatment as determined by the PLA assay. Scale bar = 20μm.

C) Quantification of PLA signal (percentage of PLA positive cells vs time) depicting the kinetics of GFP-EXD2 recruitment to nascent DNA upon HU treatment. n = 3 independent experiments, error bars represent +/- SEM.

D) Quantification of PLA signal (percentage of PLA positive cells vs time) depicting kinetics of MRE11 recruitment to nascent DNA upon HU treatment. n = 3 independent experiments, error bars represent +/- SEM.

E) Survival of U2OS control cells and U2OS *EXD2*$^{-/-}$ cells treated with the indicated doses of Mitomycin C. Survival data from two independent *EXD2*$^{-/-}$ clones are depicted. Data represent mean +/- SEM, (n=3 independent experiments).

F) Western blotting confirming EXD2 knock-out in U2OS *EXD2*$^{-/-}$ clones. Two independent clones are shown. MCM2 serves as a loading control.

G) Western blotting showing the relative levels of expression of FLAG-HA-EXD2 WT or nuclease dead (ND) mutant proteins in HeLa *EXD2*$^{-/-}$ cells stably expressing these fusion proteins. Two independent clones for each construct are shown; α-tubulin serves as a loading control.

H) Phosphor imaging of 5’ radiolabeled indicated DNA substrates (labelled strand shown in red) incubated for indicated amounts of time with EXD2 WT protein.

Figure S3 – related to Figures 5 and 6

A) Western blotting confirming the depletion of MRE11 in HeLa WT and *EXD2*$^{-/-}$ cells 72h post-transfection with control siRNA (siCtrl) or siRNA targeting MRE11 as indicated. PCNA serves as a loading control.

B) Quantification of HeLa WT and *EXD2*$^{-/-}$ cells for Rad51/Biotin PLA foci (mean, +/- SEM). Cells were treated with 4 mM HU for 3h as indicated. n=4 independent experiments, statistical significance was determined using the Mann-Whitney test (upper panel). Quantification of Rad51/Biotin PLA foci in HeLa WT cells treated with control siRNA (siCtrl) or siRNA targeting BRCA1 (siBRCA1) (bottom panel). Cells were treated with 10µM EdU for 10 min followd by 4 mM HU for 3h as indicated. Bars represent SEM, n= a minimum of 50 cells pooled from 2 independent experiments, statistical significance was determined using the Mann-Whitney test. Scale bar = 10µm.

C) Quantification of HeLa WT and *EXD2*$^{-/-}$ cells for BRCA1/Biotin PLA foci (mean, +/- SEM). Cells were treated with 4 mM HU for 3h as indicated. n=3 independent experiments, statistical significance was determined using the Mann-Whitney test.

D) Quantification of HeLa WT and *EXD2*$^{-/-}$ cells for MRE11/Biotin PLA foci (mean, +/- SEM). Cells were treated with 4 mM HU for 3h as indicated. Treatment with siRNA was carried out as indicated with control siRNA (siCtrl) or siRNA targeting BRCA1 (siBRCA1). BRCA1 knockdown is confirmed by western blotting using antibodies against BRAC1 and MCM2 which acts as a loading control. n=3 independent experiments, statistical significance was determined using the Mann-Whitney test.

E) Western blotting confirming the depletion of SMARCAL1 in HeLa WT and *EXD2*$^{-/-}$ cells 72h post-transfection with control siRNA (siCtrl) or siRNA targeting SMARCAL1 as indicated. PCNA serves as a loading control.

Figure S4 – related to Figure 6

A) Western blot confirming SMARCAL1 knockdown in U2OS WT and *EXD2*$^{-/-}$ clones. MCM2 serves as a loading control.

B) Quantification of the frequency of 53BP1 foci in U2OS WT and *EXD2*$^{-/-}$ S/G2 cells treated with control siRNA (siCtrl) or siRNA targeting SMARCAL1 (siSMARCAL1) and representative images. Cyclin A (green) acts as a marker for S/G2 cells, DAPI acts as a nuclear stain. Error bars represent +/- SEM from three independent experiments. The Mann-Whitney test was used to determine statistical significance. Scale bar = 20μm.

C) Quantification of the U2OS WT and *EXD2*$^{-/-}$ cells transfected with either control siRNA (siCtrl) or siRNA targeting SMARCAL1 (siSMARCAL1) showing micronuclei and representative images. Error bars represent +/- SEM from three independent experiments. The student's t-test was used to determine statistical significance. Scale bar = 20μm.

Figure S5 – related to Figure 6

A) Western blot confirming RECQL1 knockdown in U2OS WT and *EXD2*$^{-/-}$ clones. MCM2 serves as a loading control.

B) Quantification of the frequency of 53BP1 foci in HeLa WT and *EXD2*$^{-/-}$ S/G2 cells treated with control siRNA (siCtrl) or siRNA targeting RECQ1 (siRECQ1) and representative images. Cyclin A (green) acts as a marker for S/G2 cells, DAPI acts as a nuclear stain. Error bars represent +/- SEM from three independent experiments.

The Mann-Whitney test was used to determine statistical significance. Scale bar = 20μm.

C) Box plot of CldU/IdU tract ratios of HeLa WT and *EXD2*$^{-/-}$ cells transfected with either control siRNA or siRNA targeting RECQ1. IdU/CldU pulse labelling was followed by treatment with 4mM HU for 4 h. Whiskers indicate 5-95 percentile, n ≥ 100 tracts pooled from two independent experiments. Statistical significance was determined using the Mann-Whitney test.

D) Quantification of the HeLa WT and *EXD2*$^{-/-}$ cells transfected with either control siRNA (siCtrl) or siRNA targeting RECQ1 (siRECQ1) showing micronuclei and representative images. Error bars represent +/- SEM from three independent experiments. The student's t-test was used to determine statistical significance. Scale bar = 20μm.

E) Box plot of CldU/IdU tract ratios of HeLa WT and *EXD2*$^{-/-}$ cells untreated or pre-treated with MRE11 inhibitor Mirin (50μM). After IdU pulse cells were exposed to 2mM HU for 2h followed by release to CldU pulse for 60 minutes. Whiskers indicate 5-95 percentile, n ≥ 150 tracts pooled from two independent experiments. Statistical significance was determined using the Mann-Whitney test.

F) Quantification of PARP1/Biotin PLA foci in HeLa WT and *EXD2*$^{-/-}$ clones treated with control siRNA (siCtrl) or siRNA targeting BRCA1 (siBRCA1). Cells were treated with 10μM EdU for 10 min followd by 4 mM HU for 3h as indicated. Bars represent SEM, n= a minimum of 50 cells pooled from 2 independent experiments, statistical significance was determined using the Mann-Whitney test. Western blot confirming BRCA1 knockdown in HeLa control cells and *EXD2*$^{-/-}$ clones. MCM2 serves as a loading control. Scale bar = 10μm.

G) Schematic model of the combined roles of EXD2 and RECQ1 in counteracting fork regression. RECQ1-dependent helicase activity remodels the regressed fork providing a substrate for EXD2 nuclease. EXD2-dependent processing of the regressed nascent strand promotes efficient fork restart.

Figure S6 related to Figures 6 and 7

A) 5' radiolabeled indicated DNA substrates (3 nM molecules, labelled strand shown in red, length of the regressed arm indicated) were incubated for indicated amounts of time with EXD2 WT protein (25 nM). Samples were resolved on a on a 20% TBE-Urea polyacrylamide gel (bottom panel) and visualised by phosphorimaging.

B) 5' radiolabeled indicated DNA substrate (3 nM molecules, labelled strand shown in red) was incubated for indicated amounts of time with EXD2 WT or nuclease dead (ND) protein (25 nM) as indicated. Samples were resolved on a on a 20% TBE-Urea polyacrylamide gel (bottom panel) and visualised by phosphorimaging.

C) Quantification of the disappearance of indicated full length substrates from Fig. 6G.

D) Western blotting confirming the depletion of BRCA1 in HeLa WT and *EXD2*$^{-/-}$ cells 72h post-transfection with control siRNA (siCtrl) or siRNA targeting BRCA1 as indicated. α-tubulin serves as a loading control.

E) Western blotting confirming the depletion of BRCA2 in HeLa WT and *EXD2*$^{-/-}$ cells 72h post-transfection with control siRNA (siCtrl) or siRNA targeting BRCA2 as indicated. α-tubulin serves as a loading control.

F) Western blotting confirming the depletion of BRCA1 in U2OS WT and *EXD2*$^{-/-}$ cells 72h post-transfection with control siRNA (siCtrl) or siRNA targeting BRCA1. MCM2 acts as a loading control.

G) Western blotting confirming the depletion of EXD2 in DLD1 WT and DLD1 *BRCA2$^{-/-}$* cells 72h post-transfection with control siRNA (siCtrl) or siRNA targeting EXD2. MCM2 acts as a loading control.

H) Western blotting confirming the depletion of EXD2 in SUM149 and SUM149 revertant cells 72h post-transfection with control siRNA (siCtrl) or siRNA targeting EXD2. MCM2 acts as a loading control.

I) Western blotting confirming the depletion of BRCA1 in RPE1 WT, *EXD2$^{-/-}$* and *EXD2$^{ND/ND}$* cells 72h post-transfection with control siRNA (siCtrl) or siRNA targeting BRCA1. MCM2 acts as a loading control.

Figure S7 related to Figure 7

A) Box plot of CldU/IdU tract ratios of HeLa WT and *EXD2$^{-/-}$* cells transfected with either control siRNA or siRNA targeting either BRCA1 (left panel) or BRCA2 (right panel). IdU/CldU pulse labelling was followed by treatment with 4mM HU for 4 h. Whiskers indicate 5-95 percentile, $n \geq 100$ tracts pooled from two independent experiments. Statistical significance was determined using the Mann-Whitney test.

B) Box plot of CldU tract length ratios of associated sister forks from HeLa WT and *EXD2$^{-/-}$* cells transfected with either control siRNA or siRNA targeting BRCA1. Whiskers indicate 5-95 percentile, $n \geq 40$ sister fork pairs pooled from 2 independent experiments. Statistical significance was determined using the Mann-Whitney test.

C) Quantification of the frequency of dicentric chromosomes from mitotic spreads from U2OS WT and *EXD2$^{-/-}$* cells (mean +/- SEM, n=50 metaphase spreads pooled from 2 independent experiments). Representative images of analysed metaphase spreads.

D) Quantification of the relative alt-EJ efficiency in cells treated with control siRNA or siRNA targeting EXD2 as indicated (mean, n=2 independent experiments).

E) Western blotting confirming the depletion of EXD2 and MRE11 in U2OS EJ2-GFP cells 72h post-transfection with control siRNA (siCtrl) or siRNA targeting EXD2 or MRE11 as indicated. PCNA serves as a loading control.

Table S1 – related to STAR method section: *In vitro* nuclease assay.

DNA oligonucleotides used in *in vitro* nuclease assays.

A

Protein	No. of Peptides
PCNA	13
Pol delta sub. p66	7
Pol delta sub. p50	9
Pol delta cat. subunit	30
Pol alpha sub. B	6
Pol alpha cat subunit	16
RFC1	12
RFC2	8
RFC3	12
RFC4	18
RFC5	9
MCM2	28
MCM3	30
MCM4	34
MCM5	28
MCM6	26
MCM7	33
RPA1	21
RPA2	6
RPA3	3
PSF3	5
SAMHD1	5
**EXD2**	**5**

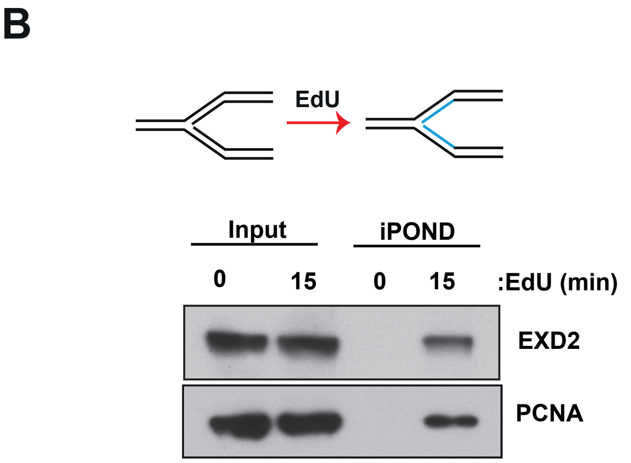

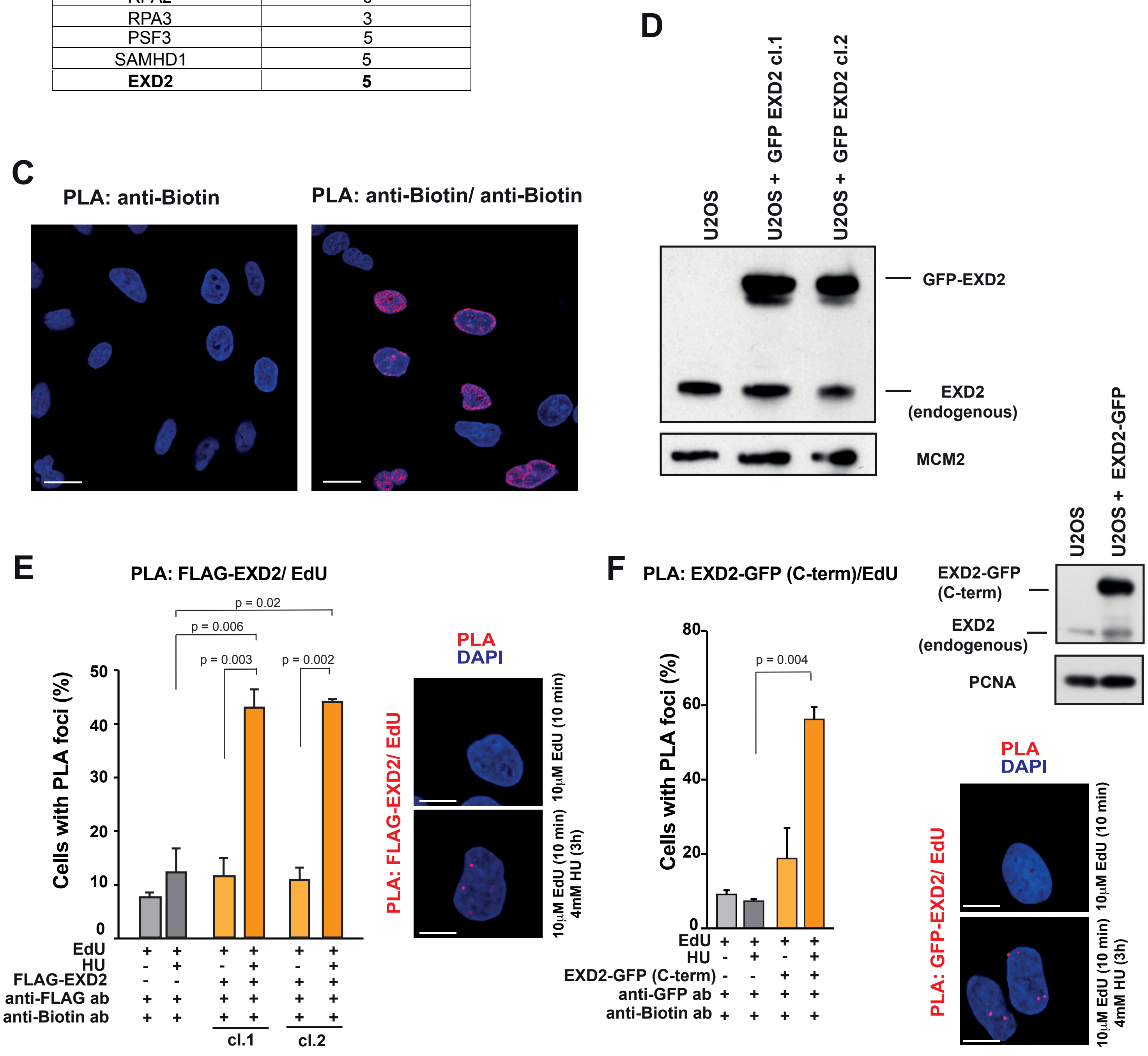

Figure S1

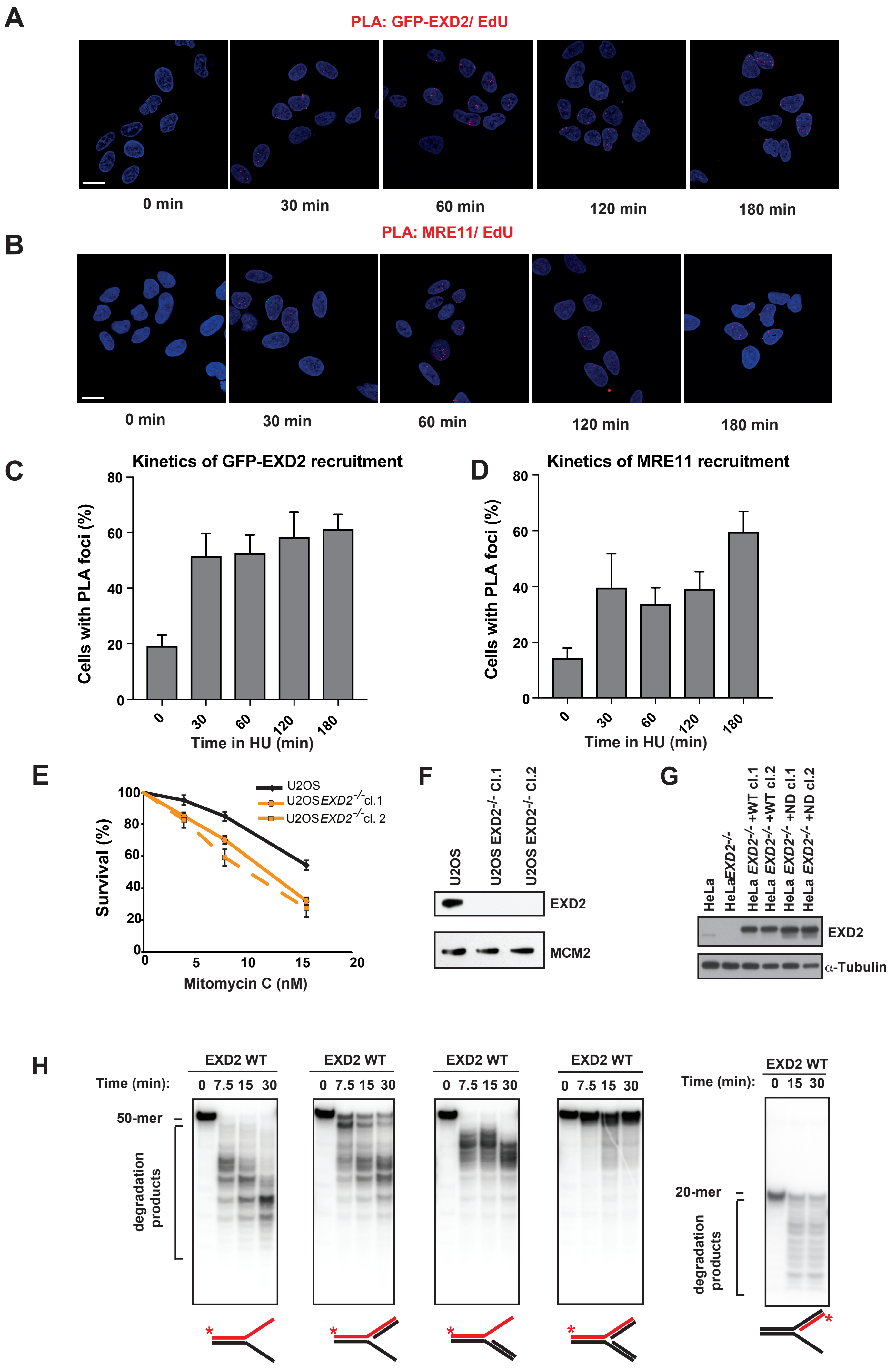
A
PLA: GFP-EXD2/ EdU
0 min
30 min
60 min
120 min
180 min
B
PLA: MRE11/ EdU
0 min
30 min
60 min
120 min
180 min
C
Kinetics of GFP-EXD2 recruitment
Cells with PLA foci (%)
Time in HU (min)
D
Kinetics of MRE11 recruitment
Cells with PLA foci (%)
Time in HU (min)
E
U2OS
U2OS EXD2-/- cl.1
U2OS EXD2-/- cl. 2
Survival (%)
Mitomycin C (nM)
F
U2OS
U2OS EXD2-/- Cl.1
U2OS EXD2-/- Cl.2
EXD2
MCM2
G
HeLa
HeLa EXD2-/-
HeLa EXD2-/- +WT cl.1
HeLa EXD2-/- +WT cl.2
HeLa EXD2-/- +ND cl.1
HeLa EXD2-/- +ND cl.2
EXD2
α-Tubulin
H
EXD2 WT
Time (min):
0 7.5 15 30
50-mer
degradation products
20-mer

Figure S2

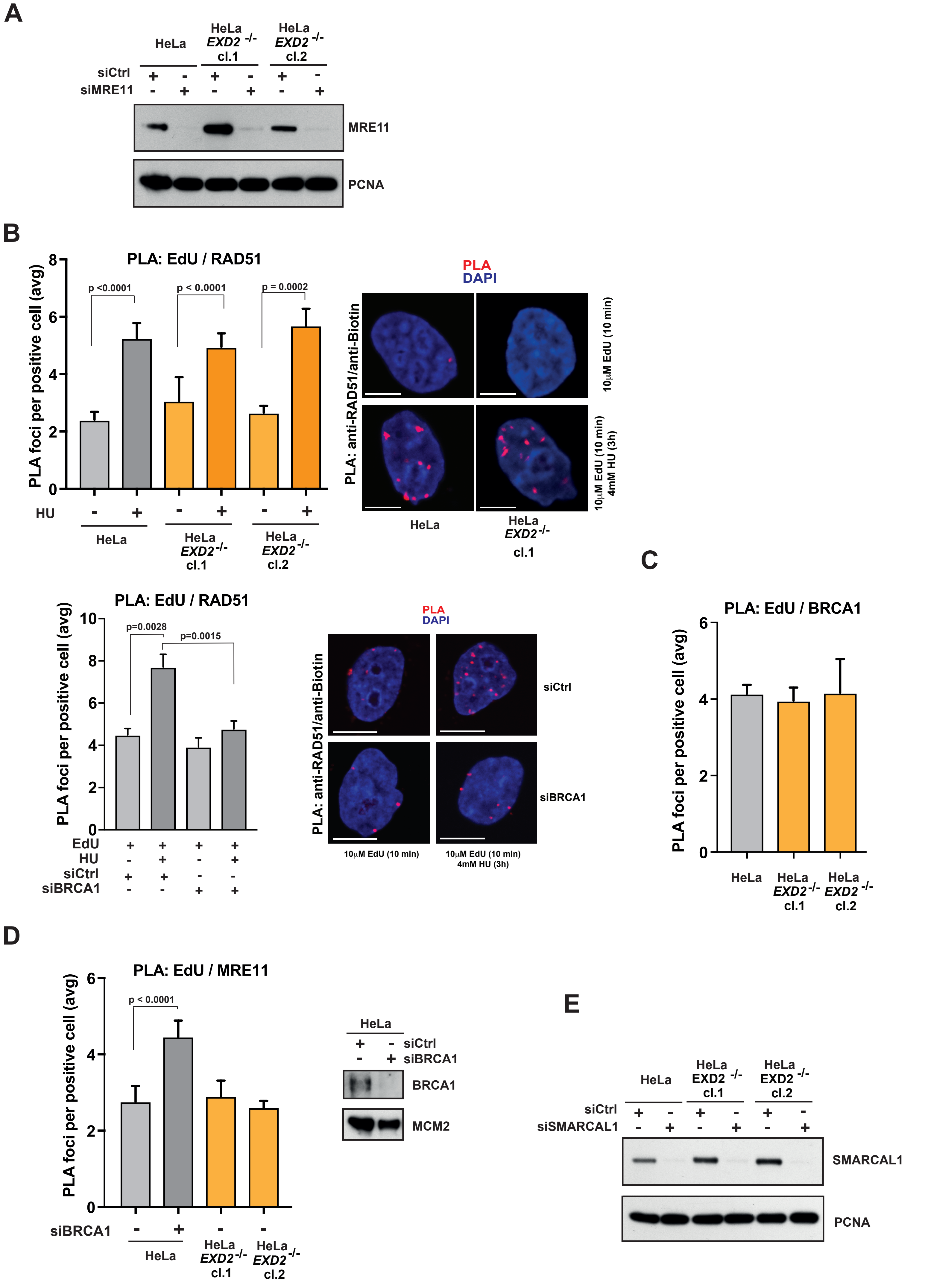
A
HeLa
HeLa EXD2 -/- cl.1
HeLa EXD2 -/- cl.2
siCtrl + - + - + -
siMRE11 - + - + - +
MRE11
PCNA
B
PLA: EdU / RAD51
PLA foci per positive cell (avg)
p <0.0001
p < 0.0001
p = 0.0002
HU - + - + - +
HeLa
HeLa EXD2-/- cl.1
HeLa EXD2-/- cl.2
PLA
DAPI
PLA: anti-RAD51/anti-Biotin
10μM EdU (10 min)
10μM EdU (10 min) 4mM HU (3h)
HeLa
HeLa EXD2-/- cl.1
PLA: EdU / RAD51
PLA foci per positive cell (avg)
p=0.0028
p=0.0015
EdU + + + +
HU - + - +
siCtrl + + - -
siBRCA1 - - + +
PLA
DAPI
PLA: anti-RAD51/anti-Biotin
siCtrl
siBRCA1
10μM EdU (10 min)
10μM EdU (10 min) 4mM HU (3h)
C
PLA: EdU / BRCA1
PLA foci per positive cell (avg)
HeLa
HeLa EXD2 -/- cl.1
HeLa EXD2 -/- cl.2
D
PLA: EdU / MRE11
PLA foci per positive cell (avg)
p < 0.0001
siBRCA1 - + - -
HeLa
HeLa EXD2-/- cl.1
HeLa EXD2-/- cl.2
HeLa
+ - siCtrl
- + siBRCA1
BRCA1
MCM2
E
HeLa
HeLa EXD2 -/- cl.1
HeLa EXD2 -/- cl.2
siCtrl + - + - + -
siSMARCAL1 - + - + - +
SMARCAL1
PCNA

Figure S3

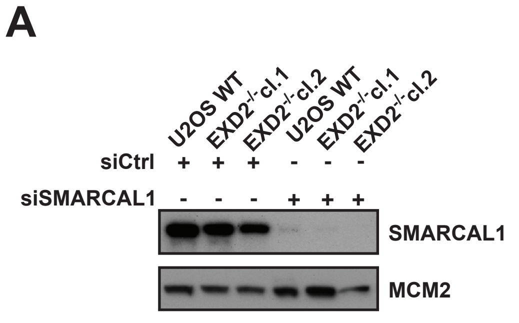
A
U2OS WT
EXD2-/- cl.1
EXD2-/- cl.2
U2OS WT
EXD2-/- cl.1
EXD2-/- cl.2
siCtrl
siSMARCAL1
SMARCAL1
MCM2

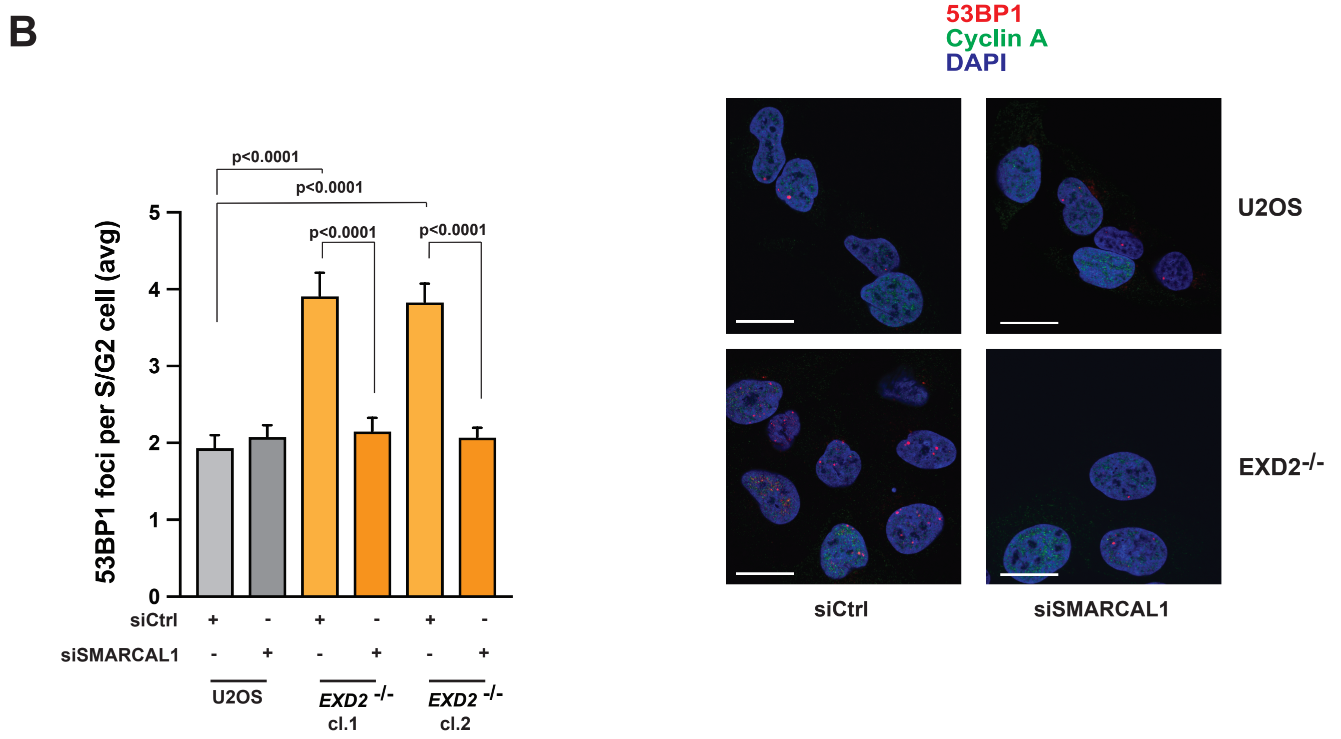
B
53BP1
Cyclin A
DAPI
53BP1 foci per S/G2 cell (avg)
p<0.0001
p<0.0001
p<0.0001
p<0.0001
siCtrl
siSMARCAL1
U2OS
EXD2 -/- cl.1
EXD2 -/- cl.2
U2OS
EXD2-/-
siCtrl
siSMARCAL1

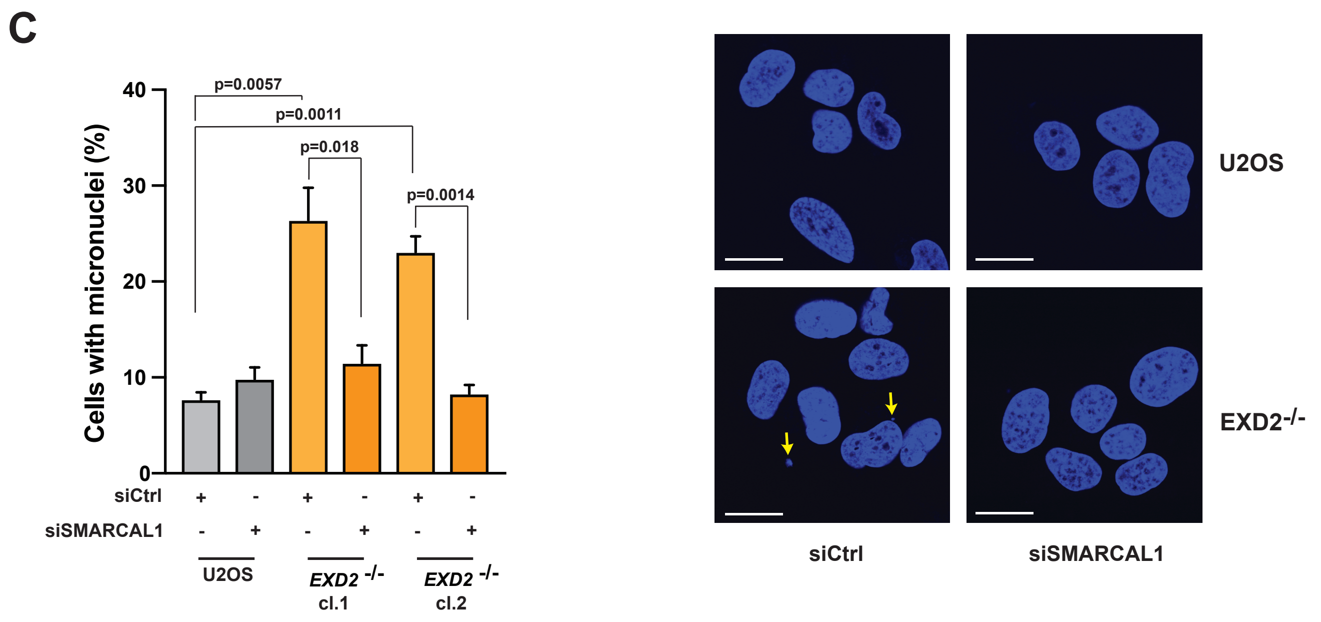
C
Cells with micronuclei (%)
p=0.0057
p=0.0011
p=0.018
p=0.0014
siCtrl
siSMARCAL1
U2OS
EXD2 -/- cl.1
EXD2 -/- cl.2
U2OS
EXD2-/-
siCtrl
siSMARCAL1

Figure S4

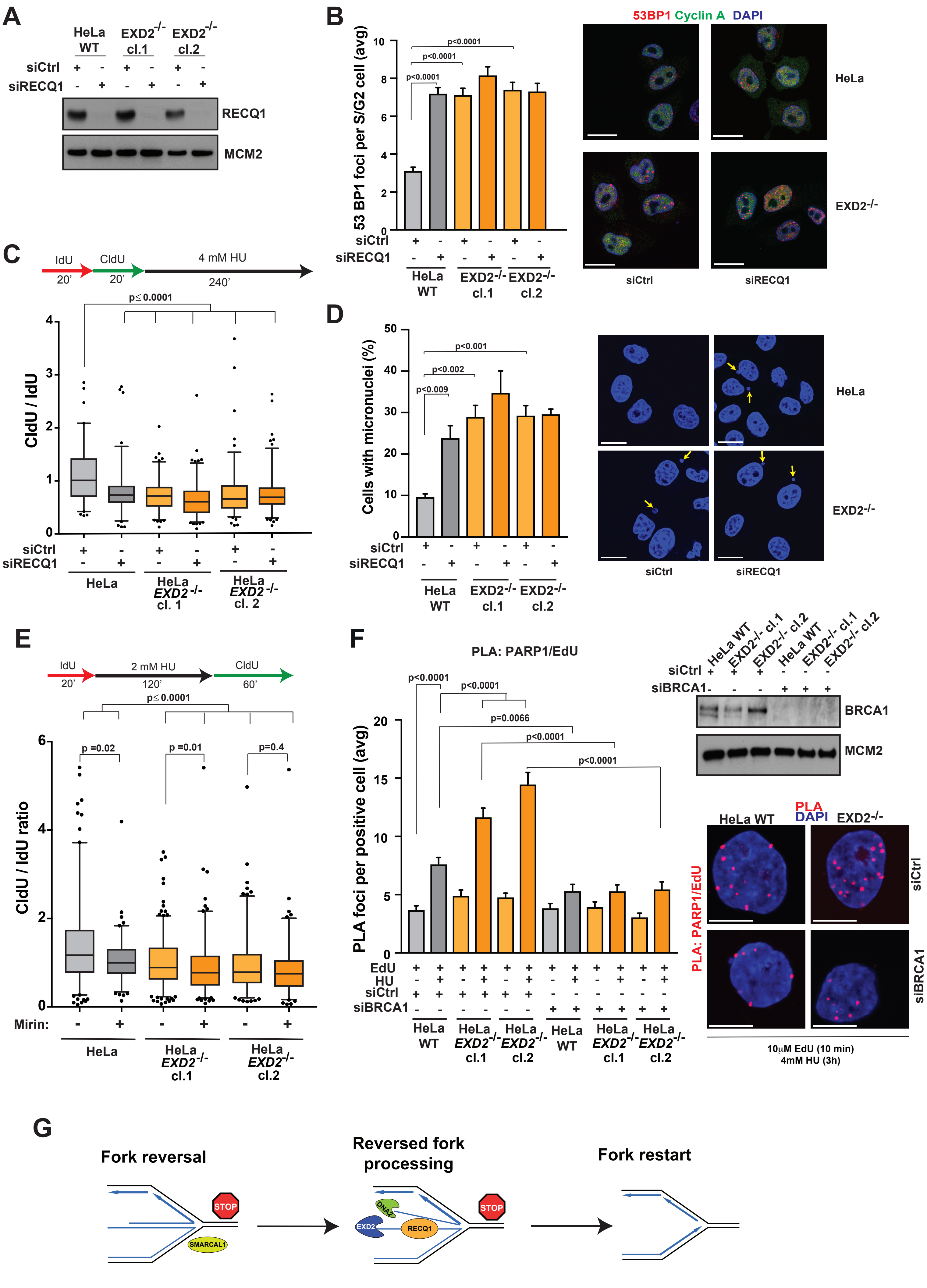

A
HeLa WT
EXD2-/- cl.1
EXD2-/- cl.2
siCtrl
siRECQ1
RECQ1
MCM2
B
53BP1 Cyclin A DAPI
53 BP1 foci per S/G2 cell (avg)
p<0.0001
p<0.0001
p<0.0001
HeLa
EXD2-/-
siCtrl
siRECQ1
C
IdU
CldU
4 mM HU
20'
20'
240'
p≤ 0.0001
CldU / IdU
HeLa
HeLa EXD2-/- cl. 1
HeLa EXD2-/- cl. 2
D
Cells with micronuclei (%)
p<0.001
p<0.002
p<0.009
E
IdU
2 mM HU
CldU
20'
120'
60'
p≤ 0.0001
p =0.02
p =0.01
p=0.4
CldU / IdU ratio
Mirin:
F
PLA: PARP1/EdU
PLA foci per positive cell (avg)
p<0.0001
p=0.0066
EdU
HU
siBRCA1
HeLa WT
EXD2-/- cl.1
EXD2-/- cl.2
BRCA1
MCM2
PLA
DAPI
PLA: PARP1/EdU
10μM EdU (10 min)
4mM HU (3h)
G
Fork reversal
Reversed fork processing
Fork restart
STOP
SMARCAL1
DNA2
EXD2
RECQ1

Figure S5

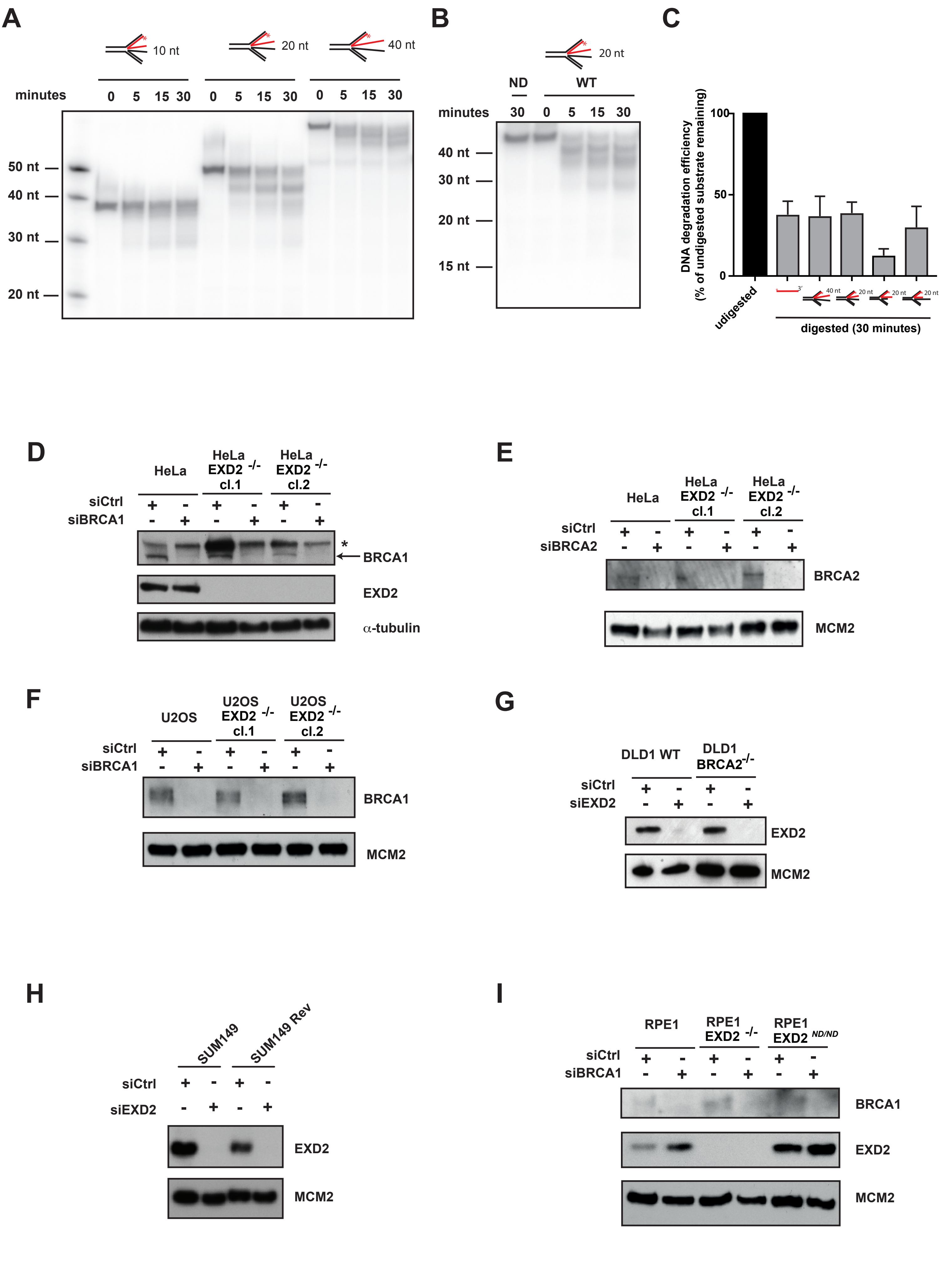
A
10 nt
20 nt
40 nt
minutes
0 5 15 30
50 nt
40 nt
30 nt
20 nt
B
20 nt
ND
WT
minutes
30 0 5 15 30
40 nt
30 nt
20 nt
15 nt
C
DNA degradation efficiency
(% of undigested substrate remaining)
100
50
0
udigested
digested (30 minutes)
D
HeLa
HeLa EXD2 -/- cl.1
HeLa EXD2 -/- cl.2
siCtrl
siBRCA1
BRCA1
EXD2
α-tubulin
E
HeLa
HeLa EXD2 -/- cl.1
HeLa EXD2 -/- cl.2
siCtrl
siBRCA2
BRCA2
MCM2
F
U2OS
U2OS EXD2 -/- cl.1
U2OS EXD2 -/- cl.2
siCtrl
siBRCA1
BRCA1
MCM2
G
DLD1 WT
DLD1 BRCA2-/-
siCtrl
siEXD2
EXD2
MCM2
H
SUM149
SUM149 Rev
siCtrl
siEXD2
EXD2
MCM2
I
RPE1
RPE1 EXD2 -/-
RPE1 EXD2 ND/ND
siCtrl
siBRCA1
BRCA1
EXD2
MCM2

Figure S6

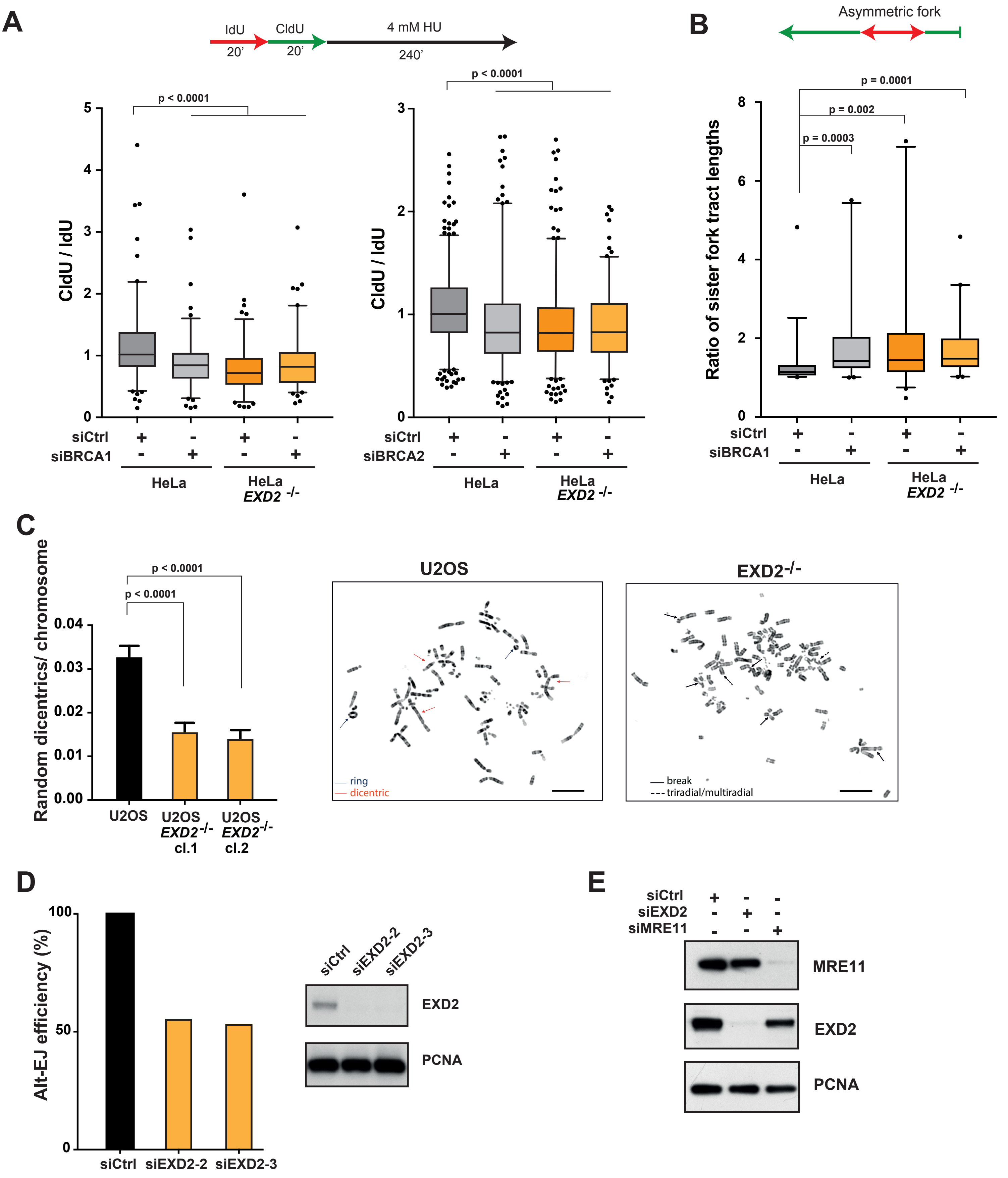
A
IdU
20'
CldU
20'
4 mM HU
240'
p < 0.0001
CldU / IdU
siCtrl
siBRCA1
HeLa
HeLa EXD2 -/-
p < 0.0001
CldU / IdU
siCtrl
siBRCA2
HeLa
HeLa EXD2 -/-
B
Asymmetric fork
p = 0.0001
p = 0.002
p = 0.0003
Ratio of sister fork tract lengths
siCtrl
siBRCA1
HeLa
HeLa EXD2 -/-
C
p < 0.0001
p < 0.0001
Random dicentrics/ chromosome
U2OS
U2OS EXD2-/- cl.1
U2OS EXD2-/- cl.2
U2OS
ring
dicentric
EXD2-/-
break
triradial/multiradial
D
Alt-EJ efficiency (%)
siCtrl
siEXD2-2
siEXD2-3
siCtrl
siEXD2-2
siEXD2-3
EXD2
PCNA
E
siCtrl
siEXD2
siMRE11
MRE11
EXD2
PCNA

Figure S7

Table S1

**Name**	**Sequence**	**Substrate** (* indicates labeled strand)
BS40	ATAAATATTTTTATTAATAATAGATCACCTTTCTTTCTCTTCTCCCCTT	split arm*, fork ds leading*, fork ds lagging*, fork ds both* [Fig.S2H left panels; right panel - not labeled]
BS41	TTCCCCTCCTCTCCTTCCTTCCTGATCTATTATTAATAAAAAATATTTAT	split arm, fork ds leading, fork ds lagging, fork ds both [Fig. S2H]
UA2	AAGGGGAGAAGAGAAAGAAAGG	fork ds leading, fork ds both [Fig. SH left panels]
UA6	GGAAGGAAGGAGAGGAGGGGAA	fork ds lagging, fork ds both
UA2_20	TTCTTTCTCTTCTCCCCTT	fork ds leading * [Fig. S2H right panel only]
RF_A	ATCTGATTCGTAGTGGACGGAGCATCCCTTTCGGCATACCATTTT;	reversed fork, double stranded* [Figure 6G and S6A and B]
RF_B	ACTCAGGCTTTACTTGGTATTTCCGCCGTCCACTACGAATCAGAT;	reversed fork [Figure 6G and S6A and B]
RF_C	AAAATGGTATGCCGAAAGGGATGCTGAAATTGAGAACGAAAAGCTGCGCCGGGAGGTTGAAGACT;	reversed fork*, single stranded* [Figure 6G and S6A]
RF_D	CGGAAATACCAAGTAAAGCCTGAGT;	reversed fork [[Figure 6G]
RF_F	CAAGCCGCCTACCTAGAACATTGTGATAGATGAAGCCTGTCGGAAATACCAAGTAAAGCCTGAGT;	reversed fork [Figure 6G and S6A and B]
RF_G	AGTCTTCAACCTCCCGGCGCAGCTTTTCGTTCTCAATTTCCGGAAATACCAAGTAAAGCCTGAGT;	reversed fork [Figure 6G]
RF_H	AAAATGGTATGCCGAAAGGGATGCTCCGTCCACTACGAATCAGAT	double stranded [Figure 6G]
RF_C_45	AAAATGGTATGCCGAAAGGGATGCTGAAATTGAGAACGAAAAGCT	reversed fork*, [Figure 6G and S6A and B]
RF_C_35	AAAATGGTATGCCGAAAGGGATGCTGAAATTGAGA	reversed fork, [Figure 6G and S6A]
RF_F_45	TTGTGATAGATGAAGCCTGTCGGAAATACCAAGTAAAGCCTGAGT	reversed fork, [Figure 6G and S6A and B]
RF_F_35	TGAAGCCTGTCGGAAATACCAAGTAAAGCCTGAGT	reversed fork, [Figure S6A]
RF_G_35	TCTCAATTTCCGGAAATACCAAGTAAAGCCTGAGT	reversed fork, [Figure 6G]
